# Fibrocyte-like cells mediate acquired resistance to anti-angiogenic therapy with bevacizumab

**DOI:** 10.1038/ncomms9792

**Published:** 2015-12-04

**Authors:** Atsushi Mitsuhashi, Hisatsugu Goto, Atsuro Saijo, Van The Trung, Yoshinori Aono, Hirokazu Ogino, Takuya Kuramoto, Sho Tabata, Hisanori Uehara, Keisuke Izumi, Mitsuteru Yoshida, Hiroaki Kobayashi, Hidefusa Takahashi, Masashi Gotoh, Soji Kakiuchi, Masaki Hanibuchi, Seiji Yano, Hiroyasu Yokomise, Shoji Sakiyama, Yasuhiko Nishioka

**Affiliations:** 1 Department of Respiratory Medicine and Rheumatology, Institute of Biomedical Sciences, Tokushima University Graduate School, 3-18-15 Kuramoto-cho, Tokushima, Tokushima 770-8503, Japan; 2 Department of Molecular and Environmental Pathology, Institute of Biomedical Sciences, Tokushima University Graduate School, 3-18-15 Kuramoto-cho, Tokushima, Tokushima 770-8503, Japan; 3 Department of Thoracic, Endocrine Surgery and Oncology, Institute of Biomedical Sciences, Tokushima University Graduate School, 3-18-15 Kuramoto-cho, Tokushima, Tokushima 770-8503, Japan; 4 Department of Thoracic Surgery, Fukui-ken Saiseikai Hospital, Funabashi 7-1, Wadanaka-cho, Fukui, Fukui 918-8503, Japan; 5 Department of Internal Medicine, Municipal Tsuruga Hospital, 1-6-60 Mishima-cho, Tsuruga, Fukui 914-8502, Japan; 6 Department of General Thoracic, Breast and Endocrinological Surgery, Faculty of Medicine, Kagawa University, 1750-1 Ikenobe, Miki-cho, Kita-gun, Kagawa 761-0793, Japan; 7 Division of Medical Oncology, Cancer Research Institute, Kanazawa University, 13-1 Takara-machi, Kanazawa, Ishikawa 920-0934, Japan; 8 JST, CREST, Tokyo 102-0076, Japan

## Abstract

Bevacizumab exerts anti-angiogenic effects in cancer patients by inhibiting vascular endothelial growth factor (VEGF). However, its use is still limited due to the development of resistance to the treatment. Such resistance can be regulated by various factors, although the underlying mechanisms remain incompletely understood. Here we show that bone marrow-derived fibrocyte-like cells, defined as alpha-1 type I collagen-positive and CXCR4-positive cells, contribute to the acquired resistance to bevacizumab. In mouse models of malignant pleural mesothelioma and lung cancer, fibrocyte-like cells mediate the resistance to bevacizumab as the main producer of fibroblast growth factor 2. In clinical specimens of lung cancer, the number of fibrocyte-like cells is significantly increased in bevacizumab-treated tumours, and correlates with the number of treatment cycles, as well as CD31-positive vessels. Our results identify fibrocyte-like cells as a promising cell biomarker and a potential therapeutic target to overcome resistance to anti-VEGF therapy.

## Abstract (web summary)

Acquired resistance to anti-angiogenic drugs, including bevacizumab, may occur in cancer patients. In this study the authors identify in the tumour microenvironment, fibrocyte-like cells derived from the bone marrow that mediate the resistance to bevacizumab through the production of FGF2.

## Graphical abstract

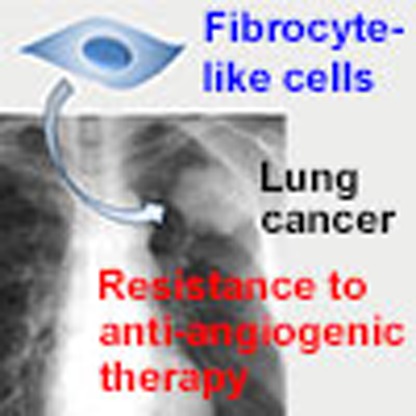

An adequate blood supply is essential for cancer cells to survive and grow, thus, the concept of inhibiting tumour angiogenesis has been applied to cancer therapy^1^,^2^. Bevacizumab is a monoclonal antibody which blocks vascular endothelial growth factor (VEGF) that is the most potent pro-angiogenic factor to mediate multiple steps of tumour angiogenesis^3^,^4^. The results from phase III clinical trials have demonstrated that the addition of bevacizumab to conventional chemotherapy improves the response rate and prolongs survival of patients with non-small cell lung cancer (NSCLC) and colon cancer^5^,^6^. However, in 2011, an announcement was made by the US Food and Drug Administration revoking the approval of bevacizumab for the treatment of metastatic breast cancer because of its insufficient efficacy and safety^7^. The possible reasons for the disappointing clinical results may include the lack of biomarkers for the efficacy of or resistance to bevacizumab treatment. A significant number of patients either do not respond to anti-VEGF agents or develop resistance to them after an initial response^8^,^9^. Therefore, it is crucial to investigate the mechanism(s) of resistance and to identify biomarkers for intrinsic and/or acquired resistance to bevacizumab treatment to develop more effective cancer therapies.

For the mechanism of the resistance to anti-VEGF therapy, the induction of hypoxia inducible factor (HIF) in tumour cells seems to be the most intensively reported. The upregulated expression of HIF in tumour cells under the hypoxic conditions initiated by the inhibition of angiogenesis induces various pro-angiogenic factors to regenerate microvessels in the tumour^2^,^8^,^10^,^11^. For host cell-mediated resistance, the involvement of tumour-associated macrophages (TAM), myeloid-derived suppressor cells (MDSC) and vascular pericytes has been reported in mice^12^,^13^,^14^,^15^,^16^. Taken together, the resistance to anti-VEGF therapy is regulated by diverse mechanisms, including those related to the tumour and host cells, although their respective functions remain incompletely understood. Moreover, the current knowledge in this field is mainly based on the observations in mouse models. Verifying the major mechanism(s) of resistance in human tumours is crucial.

In this study, we hypothesize that there are still uncovered molecular and/or cellular mechanisms that regulate the resistance to bevacizumab. To assess this hypothesis, we use mouse models of malignant pleural mesothelioma (MPM) and lung cancer, and lung cancer clinical specimens resected from patients after bevacizumab therapy to explore the mechanism of resistance to bevacizumab. We identify bone marrow-derived fibrocyte-like cells, which are double-positive for alpha-1 type I collagen and CXCR4, as a previously unrecognized cell type involved in the acquired resistance to bevacizumab via their production of fibroblast growth factor 2 (FGF2). Given that the soluble factors have not been successfully developed as a practical biomarker for the resistance to bevacizumab in clinic, fibrocyte-like cells may be a promising cell biomarker and a potential therapeutic target to overcome resistance to anti-VEGF therapy.

## Results

### Acquired resistance to bevacizumab in mouse models

**Figure 1:**
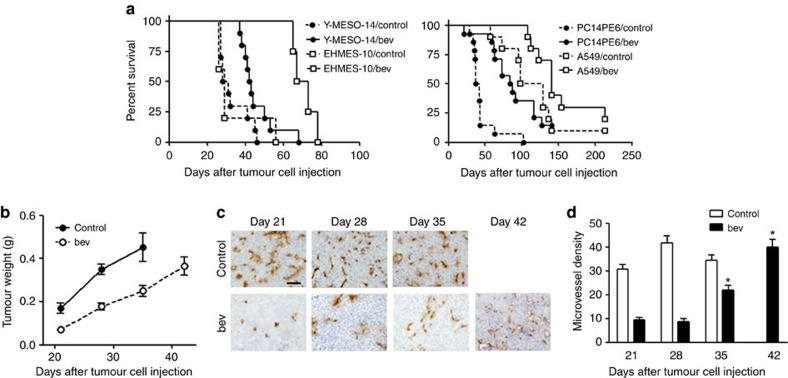
Continuous treatment with bevacizumab induces acquired resistance *in vivo*. (**a**) The survival curve of tumour-bearing mice treated with bevacizumab (bev) beginning from seven days after tumour cell injection. (left) Human MPM cell lines (Y-MESO-14 and EHMES-10) were injected orthotopically. (right) Human lung cancer cell lines (PC14PE6 and A549) were injected via the tail vein. (**b**) The weights of Y-MESO-14 intrathoracic tumours from the control and bevacizumab (bev)-treated groups at different time points. (**c**) Representative images of sections from Y-MESO-14 intrathoracic tumours stained for CD31. The tumours were harvested at different time points from the control and bevacizumab (bev)-treated groups. Scale bar, 200 μm. (**d**) Quantitative evaluation of MVD (quantified as the total number of CD31^+^ structures/HPF) in each tumour of mice (*n*=5 per group) studied in **c**. The data are shown as the means±s.e.m *P<0.05 by a one-way ANOVA.

Initially, to investigate the mechanism by which tumours develop resistance to VEGF inhibition, we orthotopically or intravenously injected immunodeficient mice with human MPM cell lines (Y-MESO-14 and EHMES-10 cells) or human lung adenocarcinoma cell lines (PC14PE6 and A549 cells) that highly express VEGF^17^,^18^,^19^,^20^. Orthotopically injected Y-MESO-14 and EHMES-10 cells produced thoracic tumours and pleural effusion, and the intravenously injected PC14PE6 cells and A549 cells produced multiple lung metastatic colonies. PC14PE6 cells also produced pleural effusion. Seven days after tumour injection, continuous treatment with bevacizumab was started. As expected, bevacizumab treatment prolonged the survival of mice injected with any of these four cell lines compared with the control group (Fig. 1a) (Y-MESO-14; *P*=0.0355, EHMES-10; *P*=0.0046, PC14PE6; *P*=0.0002, A549; *P*=0.0780, by a log-rank test). In the models of Y-MESO-14 and PC14PE6 cells we confirmed that bevacizumab effectively suppressed the tumour growth at day 28 and 35 after tumour cell inoculation, respectively (Fig. 1b and Table 1). Interestingly, the mice became moribund because of tumour progression despite continuous bevacizumab treatment (Fig. 1a,b), suggesting that the tumours exhibited resistance to bevacizumab. To investigate whether this resistance to bevacizumab was intrinsic or acquired, tumours were harvested at different time points, and the microvessel density (MVD) was immunohistochemically evaluated. MVD was significantly reduced by bevacizumab treatment; however, it gradually increase over time with continuous bevacizumab treatment (Fig. 1c,d and Supplementary Fig. 1), suggesting that the resistance to bevacizumab in these tumours was acquired. The subsequent experiments were performed using MPM cell lines, or otherwise indicated.

### Induction of stromal FGF2 in bevacizumab-resistant tumours

**Figure 2:**
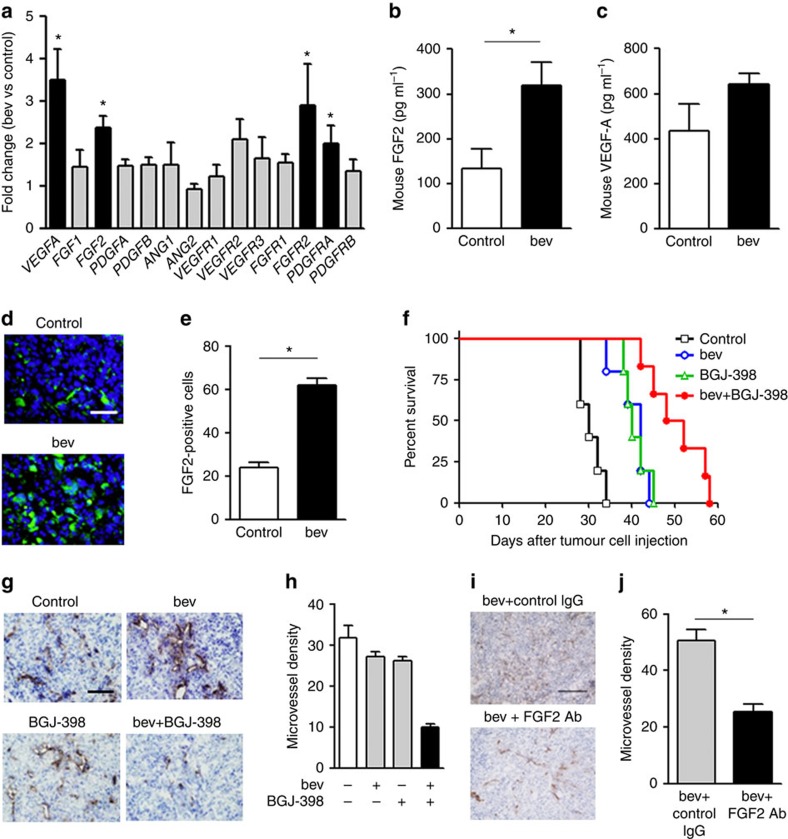
Host-derived FGF2 is upregulated in the intrathoracic Y-MESO-14 tumours resistant to bevacizumab treatment. (**a**) A comparison of mouse mRNA expression of pro-angiogenic factors in the intrathoracic Y-MESO-14 tumours treated with or without bevacizumab (bev). The fold-changes in mRNA expression of the bevacizumab-treated tumours compared with control tumours are shown. *P<0.05 by the Mann–Whitney-*U* test. The changes in the amounts of mouse (**b**) FGF2 (*n*=8 per group) and (**c**) VEGF (*n*=6 per group) in the pleural effusion of tumour (Y-MESO-14)-bearing mice treated with or without bevacizumab (bev). *P<0.05 by the Mann–Whitney-*U*-test. (**d**) Representative images of FGF2-positive cells (green) in the sections from Y-MESO-14 intrathoracic tumours treated with or without bevacizumab (bev). Scale bar, 100 μm. (**e**) The number of FGF2-positive cells in the Y-MESO-14 tumours treated with bevacizumab (bev) compared with the control group (*n*=9 per group). *P<0.01 by the Mann–Whitney-*U*-test. (**f**) The survival curves of the tumour (Y-MESO-14)-bearing mice treated with bevacizumab (bev), BGJ-398 (an FGFR inhibitor), or both. Bevacizumab was continuously given from day 7 after tumour cell injection. BGJ-398 was given from days 14–28. (**g**) Representative images of the CD31-positive vessels in the tumours harvested from each group. Scale bar, 200 μm. (**h**) Evaluation of microvessel density (MVD, quantified as the total number of CD31^+^ structures/HPF) in the tumours from each group (*n*=12 per group). *P<0.01 by a one-way ANOVA. (**i**) Representative images of the CD31-positive vessels in the tumours harvested from mice treated with bevacizumab (bev) in combination with FGF2 Ab or control IgG. Scale bar, 200 μm. (**j**) Evaluation of MVD in each group (*n*=12 per group). *P<0.01 by the Mann–Whitney-*U*-test. All data are shown as the means±s.e.m.

To identify the molecule(s) that contributes to the acquired resistance to anti-VEGF therapy, we engineered human- or mouse-specific primers to determine the differential changes in the tumour (human) and/or stromal (mouse) gene expression by performing a quantitative PCR (qPCR) analysis. The expression of tumour cell-produced pro-angiogenic ligands was examined using human-specific primers on day 28 after tumour cell (Y-MESO-14) inoculation, and the upregulation of *VEGFA* and *FGF1* was observed. However, the expression of these molecules was not changed or was under the limit of detection when the protein expression was evaluated (Supplementary Fig. 2). On the other hand, a qPCR analysis using mouse-specific primers revealed that the expression of *VEGFA*, *FGF2*, *FGFR2* and platelet-derived growth factor receptor A (*PDGFRA*) was upregulated in the bevacizumab-treated tumour stroma (Fig. 2a). As both the ligand and receptor for stromal FGF signalling were increased in the bevacizumab-treated tumours, we focused on stromal FGF2 as a candidate molecule for the acquired resistance to anti-VEGF therapy, and confirmed that the protein expression of mouse FGF2 was significantly increased in the pleural effusion of bevacizumab-treated Y-MESO-14 tumour-bearing mice (Fig. 2b). The protein level of mouse VEGF-A was also increased by 1.4-fold in the pleural effusion of bevacizumab-treated mice compared with the control mice, although it did not reach to the significance (Fig. 2c). Furthermore, an immunohistochemical analysis demonstrated that the number of FGF2-positive cells to significantly increase in bevacizumab-treated tumours compared with the control-treated tumours on day 28 after tumour cell inoculation (Fig. 2d,e).

### FGFR inhibitor improves bevacizumab resistance

To verify the role of FGF2 in bevacizumab resistance, tumour (Y-MESO-14 cells)-bearing mice were treated with a FGFR inhibitor (BGJ-398)^21^ in combination with bevacizumab. The combination therapy further prolonged the survival of mice compared with the treatment with bevacizumab or BGJ-398 alone (Fig. 2f) (control vs bevacizumab; *P*=0.0042, control vs BGJ-398; *P*=0.0017, bevacizumab vs combination; *P*=0.005, BGJ-398 vs combination; *P*=0.0078 by a log-rank test). In addition, MVD was significantly reduced by the combination treatment (Fig. 2g,h). The combination treatment of FGF2-neutralizing Ab and bevacizumab also reduced MVD in the tumour produced by Y-MESO-14 cells compared with the bevacizumab treatment in combination with control IgG (Fig. 2i,j). These results indicated that stromal FGF2 plays a role in the resistance to anti-VEGF therapy.

### Fibrocyte-like cells as FGF2-expressing cells in the tumour

**Figure 3:**
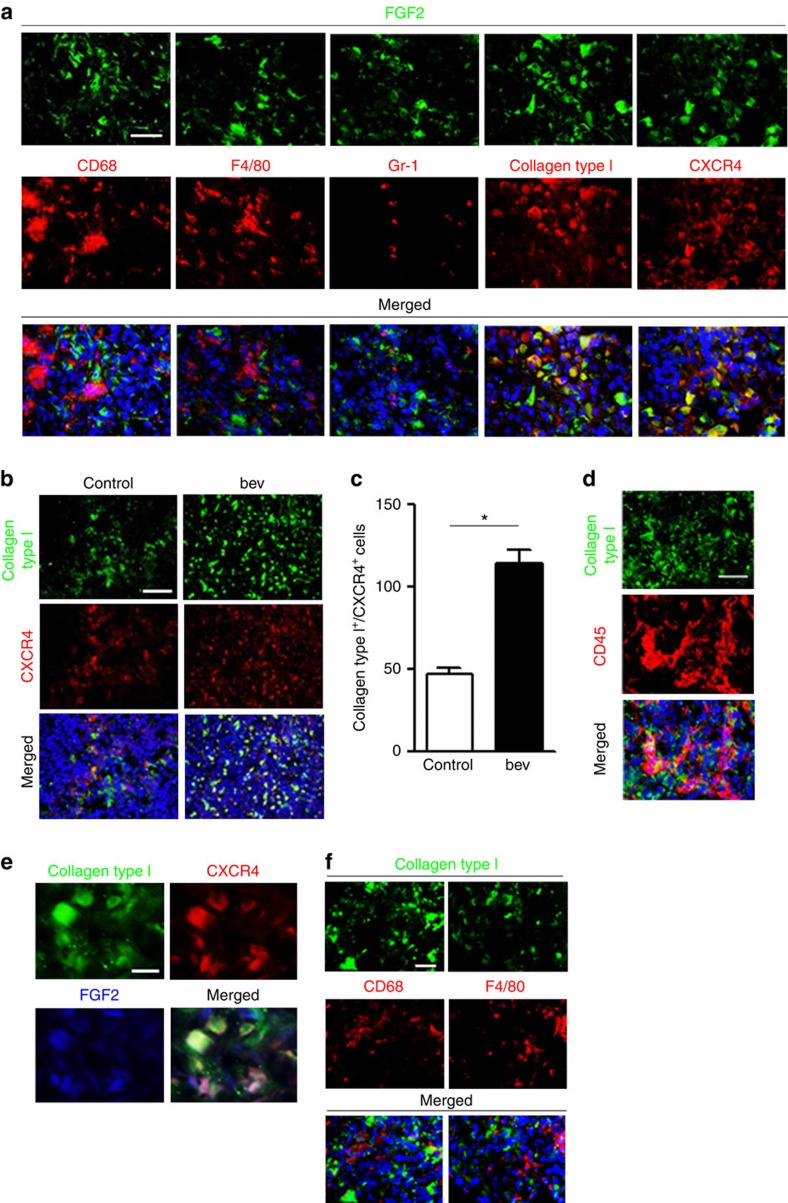
The identification of collagen type I^+^/CXCR4^+^ cells as FGF2-producing cells in the intrathoracic tumours produced by Y-MESO-14 cells. (**a**) Representative images of tumour sections from mice treated with bevacizumab. FGF2 (green) was co-stained along with CD68, F4/80, Gr-1, collagen type I or CXCR4 (red). Scale bar, 100 μm. (**b**) Double staining of collagen type I and CXCR4 in tumours from mice treated with or without bevacizumab (bev). Scale bar, 200 μm. (**c**) The number of double-positive cells in the tumours was compared between the control- and bevacizumab (bev)-treated groups (*n*=20 per group). The data are shown as the means±s.e.m. *P<0.01 by the Mann–Whitney-*U*-test. (**d**) Representative images of double staining for collagen type I and CD45 in Y-MESO-14 tumours treated with bevacizumab. Scale bar, 100 μm. (**e**) Representative images of triple staining for collagen type I, CXCR4 and FGF2 in the tumours treated with bevacizumab. Scale bar, 50 μm. (**f**) Representative images of double staining for collagen type I (green) and CD68 or F4/80 (red) in Y-MESO-14 tumours treated with bevacizumab. Scale bar, 100 μm.

We next examined what type of cells expressed FGF2 in the bevacizumab-resistant tumour environment. To identify the responsible cell type(s), we immunohistochemically assessed various cell markers (Fig. 3a and Supplementary Fig. 3a). FGF2 expression did not co-localize with CD68, F4/80 or Gr-1, suggesting that FGF2 was not expressed by macrophages or MDSCs. However, many cells did co-express FGF2 and collagen type I or CXCR4. Interestingly, collagen type I and CXCR4 were predominantly co-localized, and the number of double-positive cells was significantly increased in the bevacizumab-resistant tumours (Fig. 3b,c and Supplementary Fig. 3b,c).

The unique phenotype with the co-expression of collagen type I and CXCR4 was compatible to that of fibrocytes, which express hematopoietic progenitor cell markers (CD45, CD34) and produce extracellular matrix proteins, and also express chemokines such as CXCR4 (ref. 22). An additional immunohistochemical analysis showed that some collagen type I-positive cells also expressed CD45 (Fig. 3d). Furthermore, these collagen type I^+^/CXCR4^+^ cells were confirmed to produce FGF2 (Fig. 3e), suggesting that the collagen type I^+^/CXCR4^+^ fibrocyte-like cells play an essential role in the bevacizumab resistance of tumours by expressing FGF2. There was still a possibility that the collagen type I^+^ cells were macrophages, because macrophages may produce low levels of collagen, in addition to CXCR4 (ref. 23). However, neither CD68 nor F4/80 co-localized with collagen type I (Fig. 3f).

**Figure 4:**
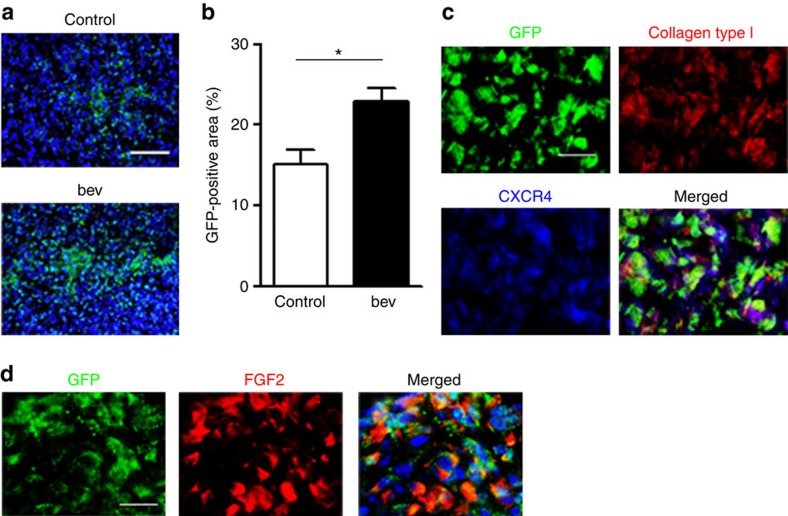
The recruitment of bone marrow-derived cells in bevacizumab-resistant tumour. Mice were given a bone marrow transplant (BMT) from GFP transgenic mice before cancer cell inoculation. (**a**) Representative images of GFP-positive bone marrow cells (green) recruited into the control or bevacizumab (bev)-treated tumours. Scale bar, 200 μm. (**b**) The percentages of GFP-positive areas in the tumours were compared between the control- and bevacizumab (bev)-treated groups (*n*=8 per group). The data are shown as the means±s.e.m. *P<0.01 by the Mann–Whitney-*U*-test. (**c**) Representative images of triple staining for GFP, collagen type I and CXCR4 in the tumours treated with bevacizumab. Scale bar, 50 μm. (**d**) Representative images of double staining for GFP and FGF2 in the tumours treated with bevacizumab. Scale bar, 100 μm.

To further confirm the characteristics of the collagen type I^+^/CXCR4^+^ fibrocyte-like cells, we sought the origin of these cells. Mice were given a bone marrow transplant (BMT) from GFP transgenic mice before cancer cell inoculation, and the collagen type I-positive cells in the tumour were assessed to determine whether they were GFP-positive. Interestingly, the number of GFP-positive cells was significantly increased in the bevacizumab-resistant tumours (Fig. 4a,b), and the collagen type I^+^/CXCR4^+^ cells were also GFP-positive (Fig. 4c), suggesting that the origin of the collagen type I^+^/CXCR4^+^ cells was the bone marrow. These results collectively indicated that the collagen type I^+^/CXCR4^+^ cells in the tumour have the distinctive features of fibrocytes. In addition, it was also confirmed that FGF2 was predominantly produced by GFP-positive cells in bevacizumab-resistant tumour in bone marrow chimeric mice (Fig. 4d).

**Figure 5:**
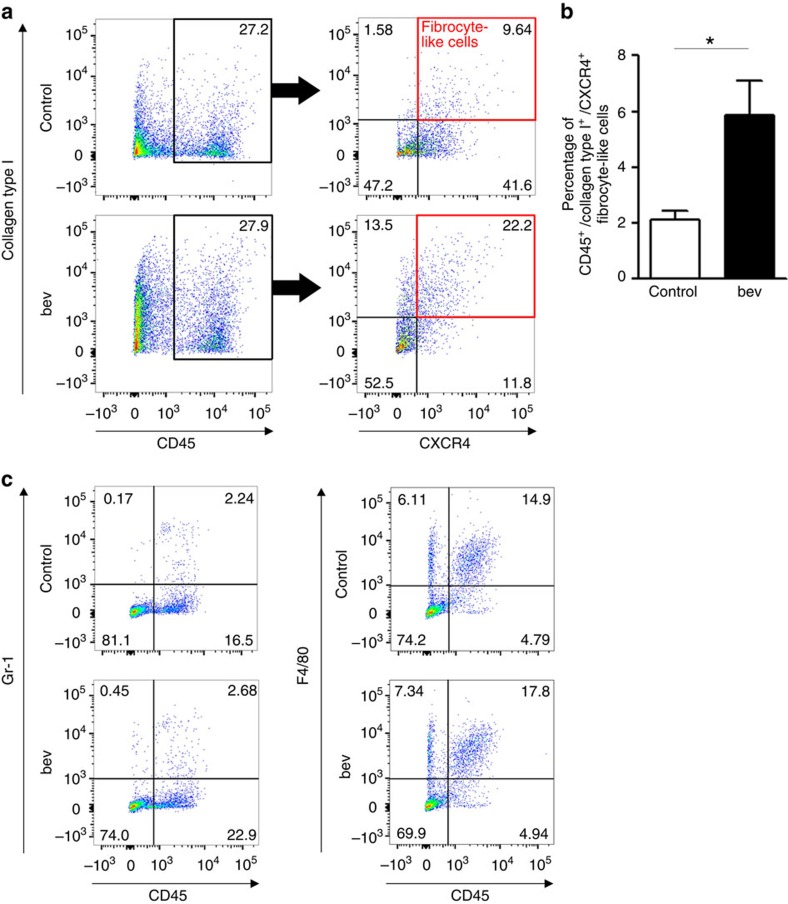
The isolation of mouse fibrocyte-like cells from Y-MESO-14 tumours by flow cytometry. (**a**) Representative analysis of the detection of fibrocyte-like cells. The tumour tissues were harvested and single-cell suspended from control- or bevacizumab (bev)-treated mice on day 28. Fibrocyte-like cells were detected as CD45^+^/collagen type I^+^/CXCR4^+^ population. Each samples contained tumour tissues from two mice. (**b**) The comparison of the percentage of CD45^+^/collagen type I^+^/CXCR4^+^ cells between control- and bevacizumab (bev)-treated group (*n*=3 in each group, each samples contained tumour tissues from two mice). *P<0.05 by Student's *t*-test. The data are shown as the means±s.e.m. (**c**) MDSCs or Macrophages were detected as CD45^+^/Gr-1^+^ cells or CD45^+^/F4/80^+^ cells, respectively. Each samples contained tumour tissues from two mice. Data are the representative of two experiments with similar results.

We finally performed the flow cytometric analysis to confirm that the number of fibrocyte-like cells was increased in the bevacizumab-resistant tumour. Single-cell suspension of control- or bevacizumab-treated tumour was stained with CD45, collagen type I and CXCR4 to detect fibrocyte-like cells. The percentage of CD45^+^/collagen type I^+^/CXCR4^+^ cells was significantly increased in the bevacizumab-resistant tumour (Fig. 5a,b). The average number of leukocytes (CD45^+^ cells) in control- and bevacizumab-treated tumour was 1.56 × 10^4^ and 2.19 × 10^4^ cells per 1 mg of tumour tissue, respectively (not significant with Student's *t*-test), whereas the number of fibrocyte-like cells was 1.12 × 10^3^ and 3.80 × 10^3^ per 1 mg of tumour tissue, respectively (*P*<0.05 with Student's *t*-test). On the other hand, the percentage of CD45^+^/Gr-1^+^ MDSCs was not changed by bevacizumab treatment (Fig. 5c), whereas the percentage of CD45^+^/F4/80^+^ macrophages was slightly increased with bevacizumab treatment (Fig. 5c).

Since the limitation of this preclinical study is that we used immunodeficient mice that lack lymphocytes, we also employed syngeneic mouse model using B16 melanoma cells and C57BL/6 mice to verify the role of other immune cells in the acquired resistance to anti-VEGF therapy (Supplementary Fig. 4). We subcutaneously injected B16 cells to C57BL/6 mice, and treated these mice with SU5416, a VEGF receptor inhibitor, continuously from day 5 after tumour cell inoculation. Similar to the results obtained from immunodeficient mice experiment, the tumour grew and MVD was increased regardless of SU5416 treatment after the initial response (Supplementary Fig. 4a–c). These tumour regrowth were suppressed by combination treatment with SU5416 and BGJ-398 (Supplementary Fig. 4d). In this model, the fibrocyte-like cells were detected as CD45^+^/FSP-1^+^ cells, and the increased number of tumour-infiltrated CD45^+^/FSP-1^+^ cells after SU5416 treatment were confirmed by immunofluorescence (Supplementary Fig. 4e,f). The effect of SU5416 treatment on the infiltration of lymphocytes into the tumour was also examined by immunofluorescence. The numbers of tumour-infiltrated CD3- or CD19-positive cells were significantly low compared with that of fibrocyte-like cells, and did not change by SU5416 treatment (Supplementary Fig. 4g).

### Character of fibrocyte-like cells in the resistant tumour

**Figure 6:**
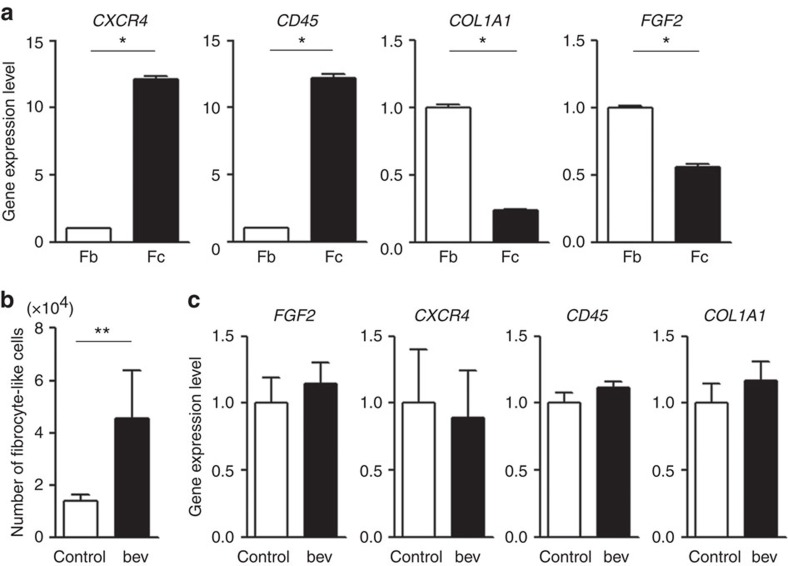
The isolation and characterization of mouse fibrocyte-like cells from normal mouse lungs or Y-MESO-14 tumours. (**a**) Fibroblasts (Fb) and fibrocyte-like cells (Fc) were isolated from the normal mouse lungs, and their mRNA expression levels were compared (*n*=3 per group). *P<0.01 by Student's *t*-test. (**b**,**c**) Fibrocyte-like cells were isolated from control- or bevacizumab (bev)-resistant tumours. (**b**) A comparison of the number of isolated fibrocyte-like cells (*n*=6 per group). (**c**) A comparison of the mRNA expression in isolated fibrocyte-like cells (*n*=6 per group). **P<0.05 by the Mann–Whitney-*U*-test. All data are shown as the means±s.e.m.

To confirm the results from immuonofluorescence and flow cytometry indicating that the number of fibrocyte-like cells was increased and that these were the FGF2-producing cells in the bevacizumab-resistant tumours, we isolated and characterized the fibrocyte-like cells from mouse tumour tissue. Before this, fibrocyte-like cells and fibroblasts were differently isolated from mouse normal lungs, and their gene expression profiles were compared. As reported previously, fibrocyte-like cells expressed higher levels of *CXCR4* and *CD45*, and relatively lower levels of *COL1A1* compared with fibroblasts (Fig. 6a)^22^. *FGF2* expression in fibrocyte-like cells was lower than that in fibroblasts. When fibrocyte-like cells were isolated from the mouse tumour tissues, the number of these cells was significantly increased in the bevacizumab-resistant tumours compared with the control-treated tumours (Fig. 6b). On the other hand, the expression of *FGF2*, as well as *CXCR4*, *CD45* and *COL1A1*, in the isolated fibrocyte-like cells was similar between the tumours (Fig. 6c), suggesting that the increase of FGF2 in bevacizumab-treated tumours was due to the increase in the number of these cells.

### Mechanism in the recruitment of fibrocyte-like cells

To investigate the mechanism(s) underlying the functions and recruitment of fibrocyte-like cells in the bevacizumab-resistant tumours, *in vitro* cell-proliferation experiments were performed using human fibrocyte-like cells derived from peripheral blood mononuclear cells. We first confirmed that bevacizumab or BGJ-398 inhibited VEGF- or FGF2-induced proliferation of human umbilical vein endothelial cells (HUVECs), respectively (Supplementary Fig. 5). As reported previously, the supernatant of fibrocyte-like cells induced the proliferation of the HUVECs^24^, but interestingly, their proliferation was inhibited only by BGJ-398, not by bevacizumab (Supplementary Fig. 5), supporting our results showing that the fibrocyte-like cells express FGF2.

**Figure 7:**
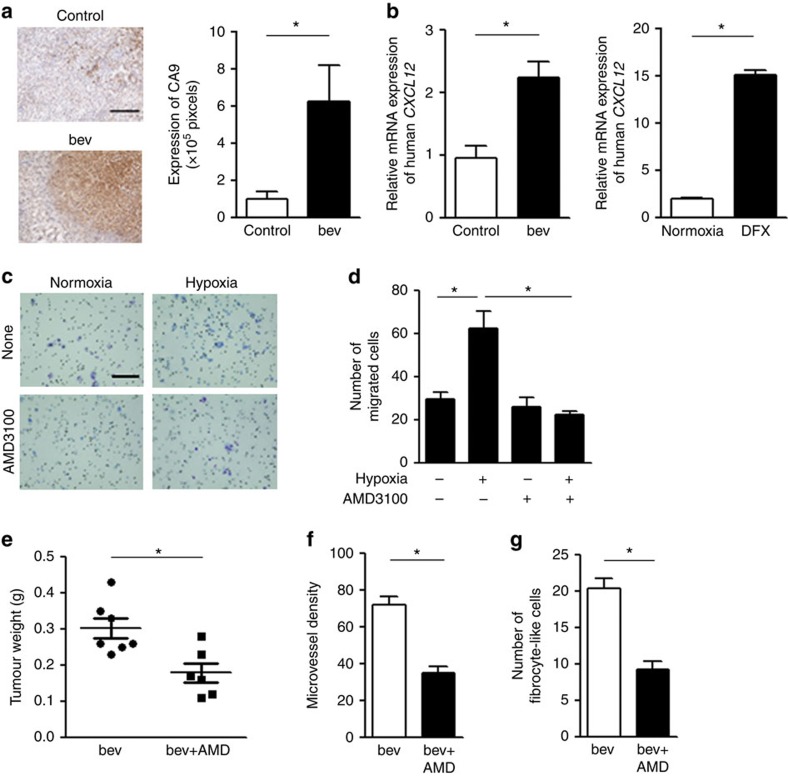
The involvement of CXCL12-CXCR4 axis in the fibrocyte-like cell-mediated angiogenesis. (**a**) Assessment of hypoxic lesion in the tumour with immunohistochemistry using anti-CA9 antibody. (left) representative images of the hypoxic lesion stained in brown in the tumour produced by Y-MESO-14 cells treated with or without bevacizumab (bev). Tumours were harvested 28 days after the tumour cell-injection. Scale bar, 200 μm. (right) Evaluation of CA9-positive area (*n*=6 per group). *P<0.05 by the Mann–Whitney-*U*-test. (**b**) (left) the relative mRNA expression of human (tumour cell) *CXCL12* in control- or bevacizumab (bev)-treated Y-MESO-14 tumour tissues (*n*=5 per group). *P<0.01 by the Mann–Whitney-*U*-test. Right, the mRNA expression of *CXCL12* in Y-MESO-14 cells cultured under normoxia or chemical hypoxia induced by deferoxamine (DFX) *in vitro* (*n*=3 per group). *P<0.01 by Student's *t*-test. (**c**) Representative images of the migrated fibrocyte-like cells *in vitro*. Fibrocyte-like cells were cultured with or without a CXCR4 inhibitor (AMD3100) in the upper chamber, and those that migrated towards the lower chamber were stained. The lower chamber contained Y-MESO-14 cells cultured under normoxia or chemical hypoxia. Scale bar, 200 μm. (**d**) The number of fibrocyte-like cells that migrated under each condition. *P<0.05 by a one-way ANOVA. All *in vitro* data are representative of duplicate experiments with similar results. (**e**–**g**) The effect of CXCR4 inhibition in addition to bevacizumab treatment *in vivo*. Thoracic tumours (Y-MESO-14) were harvested from mice treated with bevacizumab (bev) or bevacizumab in combination with AMD3100 (AMD). (**e**) A comparison of the tumour weights. (**f**) A comparison of MVD. (**f**) A comparison of the number of fibrocyte-like cells (collagen type 1^+^/CXCR4^+^ cells) (*n*=6–7 per group). *P<0.01 by the Mann–Whitney-*U*-test. All *in vivo* and *in vitro* data are shown as the means±s.e.m.

To determine the mechanism by which fibrocyte-like cells were recruited into the tumour environment, we performed human-specific gene profiling using tumour tissues (Y-MESO-14). Among the genes that were relatively upregulated in the bevacizumab-resistant tumours compared with the control tumours, we focused on *CXCL12*, the ligand of CXCR4 (Supplementary Fig. 6). Because CXCL12 is the target molecule of HIF-1α, the hypoxic condition resulting from bevacizumab treatment could lead the tumour cells to produce CXCL12, which would in turn prompt the CXCR4^+^ cells to migrate into the tumour. Indeed, the area of hypoxia was significantly increased (Fig. 7a) and the *CXCL12* expression was confirmed to be significantly upregulated in the bevacizumab-treated Y-MESO-14 tumours compared with the control tumours (Fig. 7b, left panel). *CXCL12* expression was also induced in a chemical hypoxic condition induced by treating Y-MESO-14 cells with deferoxamine (DFX) *in vitro* (Fig. 7b, right panel).

We then examined whether the CXCL12 induced by the hypoxic condition might be able to regulate the migration of fibrocyte-like cells. As expected, the number of migrated fibrocyte-like cells was strongly increased when the cells were treated with the supernatant of Y-MESO-14 cells cultured under hypoxic conditions. Moreover, this induction of migration was inhibited by a CXCR4 inhibitor (AMD3100)^25^, indicating the important role of the CXCL12-CXCR4 axis in the migration of fibrocyte-like cells (Fig. 7c,d).

To further verify the importance of the fibrocyte-like cells recruited by the CXCL12-CXCR4 axis in the development of bevacizumab resistance *in vivo*, tumour (Y-MESO-14)-bearing mice were treated with bevacizumab and/or AMD3100. The combination of AMD3100 with bevacizumab further inhibited tumour growth (Fig. 7e), angiogenesis (Fig. 7f) and fibrocyte-like cells recruitment (Fig. 7g), suggesting that CXCR4 inhibition could partially overcome the development of bevacizumab resistance by inhibiting the re-angiogenesis mediated by fibrocyte-like cells. On the other hand, AMD3100 is known to induce the mobilization of hematopoietic stem cells (HSCs) from bone marrow (BM) to the peripheral blood, mainly in the field of bone marrow transplantation in non-solid tumours^26^,^27^. Thus, since we observed that the number of fibrocyte-like cells in the tumour was inhibited by AMD3100 treatment, there is a possibility that its inhibition might be due to the reduction in the production of fibrocyte-like cells from their BM-ancestor cells. To test this hypothesis, we analysed Sca-1^+^ HSCs in the BM of bevacizumab- or bevacizumab plus AMD3100-treated mice by flow cytometry. As a result, the addition of AMD3100 in bevacizumab treatment did not affect the number of HSCs in BM (Supplementary Fig. 7) suggesting that the reduced number of fibrocyte-like cells in the tumours treated with AMD3100 was not due to the effects on BM in this model.

Ceradini *et al*.^28^ have demonstrated that HIF-1-induced CXCL12 expression increases the adhesion, migration and homing of circulating CXCR4^+^ endothelial progenitor cells to ischaemic tissue using a mouse ischaemic model. Because VEGF inhibition produces low oxygen conditions in the tumour, these results supports our data showing that bevacizumab treatment recruits fibrocyte-like cells via the CXCL12-CXCR4 axis, although other mechanisms are supposed to be involved^29^,^30^. Other than CXCR4-positive population, fibrocytes are reported to have a minor population positive of CCR7 instead of CXCR4 (ref. 30). We performed mouse-specific qPCR analysis using the tumour tissues produced by Y-MESO-14 cells, and found that the expressions of both stromal *CXCR4* and *CCR7* were increased by bevacizumab treatment, and the expression of *CCR7* was significantly lower in the tumour tissue compared with *CXCR4* expression, (Supplementary Fig. 8), suggesting that CXCR4-positive population plays a role in bevacizumab resistance. In addition, it is also possible that fibrocyte-like cells proliferate in the tumour tissue instead of recruiting from the blood stream. To confirm whether bone marrow-derived cells (BMDCs) proliferate in the tumour tissue, GFP-positive cells were co-stained with mouse-specific Ki67 using the tumour sample from BMT experiment. As a result, only 9.6% of GFP-positive cells were Ki67-positive, suggesting that the majority of fibrocyte-like cells in the tumour are recruited from the blood (Supplementary Fig. 9).

Pericytes are another important cell type to cover and protect endothelial cells from death induced by VEGF inhibition^15^,^31^,^32^. The change in pericyte coverage was also investigated immunohistochemically in our model, but we found that the pericyte coverage was not significantly increased in the bevacizumab-resistant tumours compared with the control tumours (Supplementary Fig. 10).

### Fibrocyte-like cell recruitment in human lung cancer tissue

**Figure 8:**
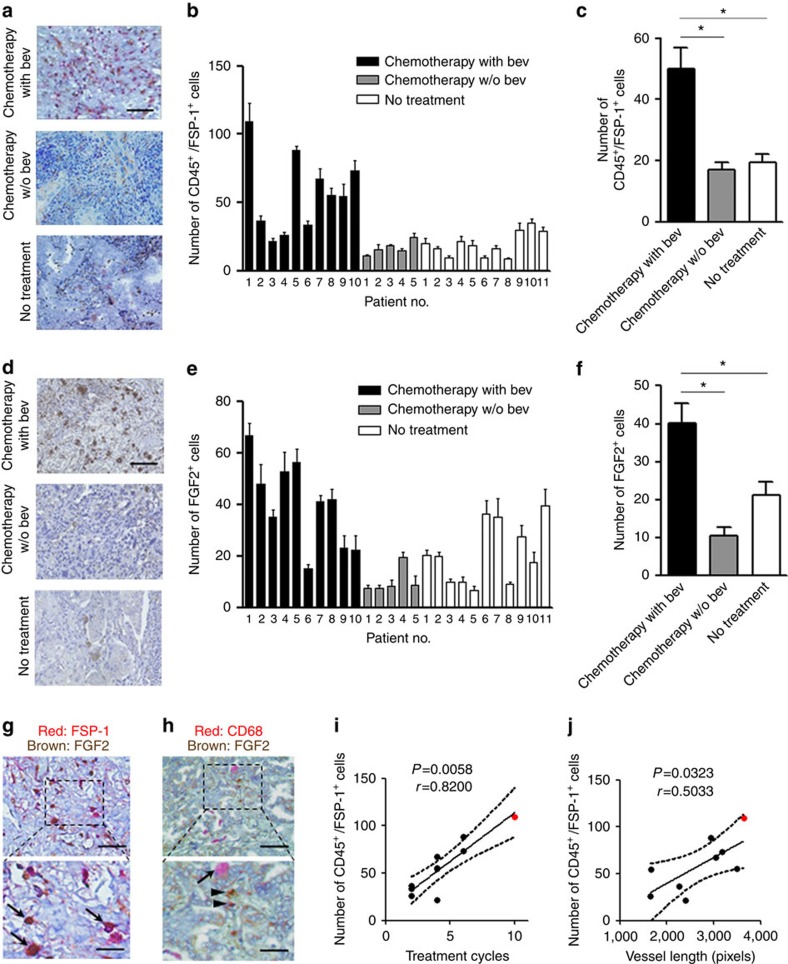
The recruitment of fibrocyte-like cells in the surgically resected human lung cancer specimens. (**a**) Representative images of fibrocyte-like cells that were double-positive for CD45 (red) and FSP-1 (brown), in the tumour tissues of patients treated with neoadjuvant chemotherapy containing bevacizumab (bev), chemotherapy without bev or with no prior therapy. Scale bar, 100 μm. (**b**) The number of CD45^+^/FSP-1^+^ cells in the individual tumour samples. (**c**) The average number of CD45^+^/FSP-1^+^ cells in each group. *P<0.01 by a one-way ANOVA. (**d**) Representative images of the FGF2-positive cells (brown) in each group. Scale bar, 100 μm. (**e**) The number of FGF2-positive cells in the individual tumour samples. (**f**) The average number of FGF2-positive cells in each group. *P<0.01 by a one-way ANOVA. (**g**,**h**) Representative images of the double staining for (**g**) FSP-1 (red) or (**h**) CD68 (red) and FGF2 (brown) in the tumour from patient no. 1. (**g**) The arrows indicate double-positive cells. (**h**) The arrow indicates a CD68 single-positive cell, and the arrowhead indicates a FGF2 single-positive cell. Scale bars, 100 μm (top), 50 μm (bottom). (**i**,**j**) The correlation of the number of CD45^+^/FSP-1^+^ cells in the tumour and (**i**) the cycles of bevacizumab used and (**j**) the vessel length in the tumour. Each dot represents an individual tumour sample from patient nos 1–10. The correlation was estimated by Spearman's correlation and a linear regression analysis (the best-fit line is indicated together with the 95% confidence bands). The red dot indicates patient no. 1, who underwent surgical resection after developing obvious clinical resistance to bevacizumab.

We finally investigated human clinical samples to assess whether there was tumour infiltration of fibrocyte-like cells in human lung cancer. Fibrocyte-like cells (CD45^+^/FSP-1^+^) were immunohistochemically detected in surgically resected tumours from lung adenocarcinoma patients. Interestingly, the number of CD45^+^/FSP-1^+^ cells was significantly higher in the tumour from patients who received bevacizumab-containing chemotherapy before the surgery compared with the tumour from the patients who received chemotherapy alone or who received no prior therapy (Fig. 8a–c). In addition, the number of FGF2-expressing cells was also significantly increased in the tumours from patients who received bevacizumab-containing chemotherapy (Fig. 8d–f). Most of the FSP-1-positive cells were also FGF2-positive, whereas the CD68-positive cells were FGF2-negative (Fig. 8g,h). Furthermore, we co-immunostained FSP-1 and CD3, CD19 or CD68 to verify the possibility that FSP-1-positive cells were lymphocytes or macrophages, and found that these markers did not co-localize with FSP-1 (Supplementary Fig. 11a). CD3-positive cells were detected to a certain extent in human lung cancer tissue although the number of these cells did not change with bevacizumab treatment (Supplementary Fig. 11b). CD19- or CD68-positive cells were minor populations regardless of bevacizumab treatment. Recently, Chung *et al*.^33^ have demonstrated that interleukin-17 (IL-17) promotes tumour resistance to VEGF inhibition by mediating immature myeloid-cell mobilization and recruitment into the tumour microenvironment in mice. The involvement of IL-17 producing cells was also investigated in our clinical samples; however, the number of IL-17-producing cells was not increased in the bevacizumab treatment group in our study (Supplementary Fig. 12). Of note, the number of CD45^+^/FSP-1^+^ cells in the bevacizumab-treated patients was significantly correlated with the number of treatment cycles of bevacizumab (Fig. 8i) and with the vessel length in the tumour (Fig. 8j). Together with the results from mouse models, these findings indicate that fibrocyte-like cells may be key regulatory cells in the tumour microenvironment that are involved in the acquisition of resistance to anti-VEGF therapy through their production of FGF2.

## Discussion

Fibrocytes, which are present in the peripheral circulation as a minor population of leukocytes, were first identified more than a decade ago^34^. Subsequent studies revealed that they are monocyte-derived cells that have features of both macrophages and fibroblasts^22^,^23^,^24^,^35^,^36^. Since there is no single specific marker for fibrocytes at present, the combination of intracellular collagen staining and the expression of a hematopoietic marker, such as CD45, plus either CD34 or CXCR4, has been considered to be a sufficiently accurate criterion for identifying fibrocytes^22^,^23^,^37^. In some studies, fibrocytes are considered as the cells double positive of CD45 and FSP-1 (refs 38, 39, 40). Based on their fibrogenic properties, fibrocytes were previously reported to be involved in the pathogenesis of various fibrotic diseases, such as pulmonary fibrosis, bronchial asthma and cardiovascular disease^41^,^42^,^43^,^44^,^45^. In the field of cancer, however, only a small number of studies have reported the existence of fibrocytes in the peripheral blood of patients^46^, and the role of fibrocytes in the pathogenesis of cancer has been unknown, and to the best of our knowledge, their role in drug resistance has not yet been examined.

Although it is complicated to determine the precise identity of monocyte-derived cell populations because of their plasticity and phenotypical overlaps, we applied the term ‘fibrocyte-like cells' for collagen type I^+^/CXCR4^+^ (and also CD45^+^ in some experiments) cells or CD45^+^/FSP-1^+^ cells, and showed those cell-mediated mechanism of the acquired resistance to bevacizumab treatment in both mouse models and human clinical tissue samples. To our knowledge, the previous studies using clinical specimens to investigate the mechanism of the resistance to anti-angiogenic therapy have been focused on the soluble factors present in peripheral blood such as VEGF, placental growth factor, CXCL12 and FGF2 (refs 47, 48, 49, 50, 51, 52). Considering that these soluble factors have not been successfully developed as a practical biomarker in clinic, and because the cell–cell interactions play crucial roles in the tumour microenvironment, it seems logical to investigate tissue samples. In our study, not all of the patients who received bevacizumab clinically acquired resistance before the surgery. However, patient no. 1, who had the highest number of fibrocytes and the highest FGF2 expression in the tumour tissue (Fig. 8b,e), underwent surgical resection after developing obvious clinical resistance to bevacizumab. This might support the involvement of fibrocyte-like cells in the resistance of human lung cancer to anti-VEGF therapy. In addition, the clinical data also suggest that the mechanism of resistance mediated by fibrocyte-like cells has already been ‘switched on' before the development of clinically relevant tumour recurrence. Although it is challenging to obtain surgically removed samples after bevacizumab treatment, given the small number of such samples available, continuous efforts are warranted to clarify the cellular mechanism by which fibrocyte-like cells may mediate the acquired resistance to anti-angiogenic therapy.

Various modes of resistance to anti-angiogenic therapy are reviewed in detail by Bergers and Hanahan^10^. According to the current knowledge, the major mechanisms of host-mediated acquired resistance include revascularization subsequent to the upregulation of alternative pro-angiogenic signals; the recruitment of pro-angiogenic cells from the bone marrow and the protection of the vasculature by pericyte coverage. Among the soluble factors that could mediate the angiogenic switch, FGF2 appears to be one of the most intensively reported factor in both the experimental and clinical settings^47^,^48^,^53^,^54^,^55^. With regard to the cells responsible for producing FGF2 in the resistant tumours, the results from our study demonstrated that fibrocyte-like cells may be a major source, in addition to macrophages and the tumour cells themselves.

Tumour angiogenesis, and possibly the re-angiogenesis that develops in the tumours resistant to anti-angiogenesis therapy, can be regulated not only by soluble pro-angiogenic factors but also by the recruitment of various BMDCs that have the capacity to regenerate the blood vessels. Those BMDCs include TAMs, TIE2^+^ monocytes, VEGFR1^+^ hemangiocytes and CD11b^+^ myeloid cells^56^,^57^,^58^,^59^,^60^,^61^. In this study, we demonstrated that the fibrocyte-like cells, but not CD3- or CD19-positive cells, in the tumour microenvironment are involved in the acquisition of resistance to anti-VEGF therapy. As previously reported^12^,^13^, TAMs do contribute on tumour angiogenesis. There is a possibility that, in addition to fibrocyte-like cells, tumour-infiltrated macrophages may also contribute on angiogenesis in our model, as the number of CD45^+^/F4/80^+^ macrophages was slightly increased with bevacizumab treatment (Fig. 5c). For this, future efforts are warranted to clarify the role of macrophages in the resistance to anti-VEGF therapy in this model. Since we observed in human clinical specimens and in immunodeficient mouse model that F4/80^+^ or CD68^+^ cells did not express FGF2 (Figs 3a and 8h), macrophages may contribute on the acquired resistance via the mechanism(s) other than FGF2-FGFR signalling.

Among the cells that could be able to contribute on the resistance to anti-angiogenesis therapy, CD11b^+^Gr1^+^ MDSCs were reported to be directly involved in the tumour refractoriness to anti-VEGF therapy in a mouse model^14^. In this study, the number of tumour-infiltrated Gr-1^+^ cells was not changed after anti-VEGF therapy. However, contribution of MDSCs on acquired resistance to anti-VEGF treatment should not be ignored, as recent reports suggest that some populations of MDSCs (CCR2^+^ or CD11c^high^/ CD14^−^/ CD123^−^) have a potential to differentiate to fibrocytes and encourage tumour progression^46^,^62^. Since these cells have not yet been clearly identified in humans, future technological developments are needed to determine whether these cells exist and are a cause of resistance in humans. There are several potential clinical implications of the present study. First, based on our preclinical data generated using mouse models, dual inhibition of FGF2 and VEGF could provide a more durable response to anti-angiogenesis therapy compared with single treatment with bevacizumab. Moreover, it is of interest to ask what other molecules (or cells) are involved after the resistance against FGF2 and VEGF. Second, fibrocyte-like cells could be the key regulator of the resistance to bevacizumab therapy. Thus, it may be instructive to target these cells to more effectively inhibit tumour angiogenesis. Third, fibrocyte-like cells could also be useful as a cellular biomarker for the resistance to bevacizumab therapy. As such, it would be interesting to develop the technology to detect these cells in the peripheral blood to predict the resistance to anti-angiogenesis therapy.

In summary, we uncovered that a cell type appears to mediate the resistance to anti-VEGF therapy. It is crucial to focus not only on the soluble factors but also on the responsible cells, to understand the mechanisms of resistance to treatment, and comprehending the mechanism mediated by fibrocyte-like cells might help to develop more effective and durable anti-angiogenesis therapy.

## Methods

### Cell lines

The human MPM cell lines, Y-MESO-14 and EHMES-10, were kindly provided by Dr Y. Sekido (Aichi Cancer Center Research Institute, Nagoya, Japan) and Dr H. Hamada (Hiroshima University Graduate School of Health Science, Hiroshima, Japan), respectively. The human NSCLC cell line PC14PE6, and mouse melanoma cell line B16 were kindly provided by Dr I.J. Fidler (M.D. Anderson Cancer Center, TX). The human NSCLC cell line, A549, was purchased from the American Type Culture Collection. Normal HUVECs were purchased from KURABO (Osaka, Japan). These cell lines were authenticated at BEX CO., LTD (Tokyo, Japan) using a Multiplex STR assay. Human thoracic tumour cell lines were maintained in RPMI 1640 medium supplemented with 10% heat-inactivated fetal bovine serum (FBS), penicillin (100 U ml^−1^) and streptomycin (50 μg ml^−1^). B16 cells were maintained in DMEM medium supplemented with 10% heat-inactivated FBS, penicillin (100 U ml^−1^) and streptomycin (50 μg ml^−1^). HUVECs were maintained in EBM-2 (Lonza, Basel, Switzerland). All cells were cultured at 37 °C in a humidified atmosphere of 5% CO_2_ in air.

### Reagents

Bevacizumab, an immunoglobulin (Ig) G1 monoclonal antibody targeting VEGF-A, was obtained from Chugai Pharmaceutical (Tokyo, Japan). For *in vivo* studies, bevacizumab was diluted in phosphate-buffered saline (PBS). BGJ-398, a FGFR-specific inhibitor, was purchased from Chemietek (Indianapolis, IN). For the *in vitro* studies, 4 mM stock solutions were prepared in DMSO. For the *in vivo* studies, BGJ-398 was formulated in PEG300/D5W (2:1). FGF2-neutralizing antibody was purchased from R&D systems (Minneapolis, MN). AMD3100, a selective CXCR4 antagonist, was purchased from Sigma (St Louis, MO). For *in vitro* studies, 10 mg ml^−1^ stock solutions were prepared in PBS. SU5416, a VEGFR-specific inhibitor, was purchased from Abcam (Cambridge, MA). For the *in vivo* studies, SU5416 was formulated in DMSO. An anti-mouse IL-2 receptor β-chain monoclonal antibody, TM-β1, was supplied by Drs M. Miyasaka and T. Tanaka (Osaka University, Osaka, Japan).

### Animals

Six-weeks-old male athymic BALB/c nude mice, severe combined immunodeficient (SCID) mice and C57BL/6 mice were obtained from Charles River Japan Inc. (Shiga, Japan) or CLEA Japan (Tokyo, Japan), respectively. Six-weeks-old male C57BL/6-BALB/c-nu/nu-EGFP mice were obtained from SLC Japan Inc. (Shizuoka, Japan). Mice were maintained under specific pathogen-free conditions throughout this study. All experiments were performed in accordance with the guidelines established by the Tokushima University Committee on Animal Care and Use. At the end of each *in vivo* experiment, the mice were anaesthetized with isoflurane and euthanized humanely by cutting the subclavian artery. All experimental protocols were reviewed and approved by the animal research committee of The University of Tokushima, Japan.

### *In vivo* orthotopic implantation models of human MPM cells

For orthotopic implantation, SCID mice were anaesthetised with tribromoethanol and had their right chest wall shaved. After sterilization of the chest wall with 70% ethanol, their right chest skin was cut, and their parietal pleura were exposed. They were then injected with Y-MESO-14 or EHMES-10 cells (1.0 × 10^6^ cells per mouse) suspended in 0.1 ml PBS into the thoracic cavity through the parietal pleura using a 27G needle^63^. To determine the therapeutic efficacy of bevacizumab against intrathoracic implanted tumour tissue, the mice were treated twice a week with 10 μg per mouse bevacizumab by intraperitoneal injection, beginning 1 week after tumour cell inoculation. The mice were killed humanely under anaesthesia, and the thoracic tumours were removed and weighed 21, 28, 35, 42 days (Y-MESO-14) or 28 and 65 days (EHMES-10) after tumour cell inoculation. Whole tumour tissues were weighted, and the weights were compared between bevacizumab-treated group and vehicle-treated group. The pleural effusion was harvested using a 1 ml syringe. To determine the effects of bevacizumab on survival, mice were treated twice a week with 10 μg per mouse bevacizumab or vehicle control from day 7 until they became moribund. To examine the effects of combination treatment with bevacizumab and BGJ-398, the mice were treated once daily with 200 μg per mouse BGJ-398 or vehicle control by oral gavage from days 21 to 35. To determine the effects of combination treatment with bevacizumab and FGF2-neutralizing antibody, the mice were treated with 20 μg per mouse goat anti-FGF2 antibody or control goat IgG (R&D systems, Minneapolis, MN) by subcutaneous injection on days 7 and 14 in combination with continuous bevacizumab treatment stated above. To examine the effects of combination treatment with bevacizumab and a CXCR4 antagonist (AMD3100), the mice were treated once daily with 5 mg kg^−1^ per mouse AMD3100 or vehicle control by subcutaneous injection from days 7 to 42. Considering the inhibitory effect of AMD3100 in the anti-tumour immune response via NK cells, all mice were pretreated with TM-β1 (1 mg per 0.3 ml PBS per mouse) by intraperitoneal injection to deplete NK cells two days before tumour inoculation^64^. The mice were killed humanely under anaesthesia, and thoracic tumours were removed on day 42. Whole tumour tissues were weighted, and the weights were compared between the groups treated with combination therapy and bevacizumab monotherapy.

### *In vivo* lung metastasis model

To establish the lung metastasis model, nude mice were intravenously inoculated via the tail vein with PC14PE6 or A549 tumour cells (1.0 × 10^6^ cells per mouse) suspended in 0.2 ml of PBS^65^. Mice were treated with bevacizumab with the same procedure used in the orthotopic models.

### GFP bone marrow chimeric mouse model

Bone marrow cells from C57BL/6-BALB/c-nu/nu-EGFP mice were extracted from the tibias and femurs and transplanted by tail-vein injection (5.0 × 10^6^ cells per mouse) into irradiated (two doses of 3.5 Gy, 3 h apart) nude mice. Three weeks after the BMT, the mononuclear cells in the peripheral blood of recipient mice were screened by Keyence BZ-9000 microscopy (Keyence, Tokyo, Japan), and were confirmed to be over 90% GFP-positive. The mice were pretreated with TM-β1 (1 mg per 0.3 ml PBS per mouse) by intraperitoneal injection to deplete NK cells three weeks after the BM transplant. Two days later, the mice were orthotopically inoculated with Y-MESO-14 tumour cells (1.0 × 10^6^ cells per mouse) and treated twice a week with 10 μg per mouse bevacizumab or vehicle control by intraperitoneal injection from day 7. The mice were killed humanely under anaesthesia and thoracic tumours were removed 28 days after tumour cell inoculation.

### *In vivo* subcutaneous implantation model of B16 cells

Mouse melanoma cell line, B16 cells (1.0 × 10^6^ cells per mouse) suspended in 0.1 ml PBS were subcutaneously inoculated to the right flank of C57BL/6 mice. Tumour size was measured by vernier caliper at least three times a week. Volume=*ab*^2^ *2*^−1^ (*a*, long diameter; *b*, short diameter). To determine the effect of SU5416 on tumour growth, the mice were treated once a day with SU5416 (5 or 10 mg per kg per mouse) or DMSO by intraperitoneal injection from day 5 until they became moribund. When the average of tumour size reached 5000–6000, mm^3^, the mice were killed humanely and the tumours were resected for the further analyses. To examine the effects of combination treatment with SU5416 and BGJ-398, the mice were treated once daily with 200 μg per mouse BGJ-398 or vehicle control by oral gavage from day 5, in addition to SU5416 treatment.

### Immunofluorescence of mouse tumour tissues

The excised tumour tissues from the model mice were placed into OCT compound (Sakura Finetechnical Co., Tokyo, Japan) and snap-frozen. Frozen tissue sections (8 μm thick) were fixed with 4% paraformaldehyde solution in PBS and used for the identification of fibrocyte-like cells using a goat anti-COL1A1 polyclonal antibody (1:100 dilution, Santa Cruz, Dallas, TX), rabbit anti-FSP-1 polyclonal antibody (1:100 dilution, Neomarkers/Lab Vision Corporation, Fremont, CA), rat anti-CXCR4 monoclonal antibody (1:100 dilution, 247506, R&D systems, Minneapolis, MN) and rat anti-CD45 monoclonal antibody (1:50 dilution, 30-F11, BD Pharmingen, Franklin Lakes, NJ). To detect macrophages, a rat anti-CD68 monoclonal antibody (1:100 dilution, ED1, Serotec, Oxford, UK) and rat anti-F4/80 monoclonal antibody (1:100 dilution, A3-1, Serotec, Oxford, UK) were used. To identify MDSCs, a rat anti-Gr-1 monoclonal antibody (1:50 dilution, RB6–8C5, BD Pharmingen, Franklin Lakes, NJ) was used. To detect T cells and B cells, a rat anti-CD3 monoclonal antibody (1:150 dilution, 17A2, BD Pharmingen, Franklin Lakes, NJ) and a rat anti-CD19 monoclonal antibody (1:150 dilution, 1D3, BD Pharmingen, Franklin Lakes, NJ) were used, respectively. To detect fibroblasts, a goat anti-αSMA polyclonal antibody (1:150 dilution, Abcam, Cambridge, MA) was used. To determine the expression of FGF2 in the above-mentioned cells, a rabbit anti-FGF2 polyclonal antibody (1:100 dilution, Santa Cruz, Dallas, TX) or goat anti-FGF2 polyclonal antibody (1:50 dilution, Sigma, St Louis, MO) were used. A rat anti-CD31/PECAM-1 monoclonal antibody (1:150 dilution, MEC 13.3, BD Pharmingen, Franklin Lakes, NJ) and rabbit anti-NG2 polyclonal antibody (1:150 dilution, Millipore, Billerica, MA) were used, respectively, to detect endothelial cells and pericytes. To detect GFP-positive cells, we used a rabbit anti-GFP monoclonal antibody (1:75 dilution, D5.1, Cell Signaling Technology, Danvers, MA) or goat anti-GFP polyclonal antibody (1:50 dilution, Santa Cruz, Dallas, TX). To detect cell proliferation, rabbit anti-mouse Ki67 antibody (1:150 dilution, D3B5, Cell Signaling Technology, Danvers, MA) was used. Alexa488, 594 and 647-labelled secondary antibodies (1:250 dilution, Invitrogen, Carlsbad, CA) were used for immunofluorescent detection. Nuclei were counterstained with DAPI (blue). In each slide, the number of positive cells was counted under a fluorescent microscope at a × 200 magnification. The pericyte coverage of the tumour vasculature was determined by performing double staining for CD31 and NG-2. The expression levels of FGF2 in the cells in the tumour microenvironment were determined by performing double staining for FGF2 and markers of tumour microenvironment cells, such as collagen type I, CXCR4, CD45, CD68, F4/80, Gr-1, CD3, CD19 or αSMA, and double positive cells were evaluated at a × 400 magnification. The expression of FGF2 in fibrocyte-like cells was determined by performing triple staining for FGF2, collagen type I and CXCR4 at a × 800 magnification. In the GFP bone marrow chimeric mouse model, the percentages of the GFP-positive areas were analysed using the Image J software program (National Institutes of Health, Bethesda, MD) at a × 200 magnification. To determine the bone marrow-derived fibrocyte-like cells, triple staining of collagen type I, CXCR4 and GFP was performed at a × 800. The expression levels of FGF2 or cell proliferation in BMDCs were determined by performing double staining for FGF2 or Ki67 and GFP at a × 400. These images were acquired by an Olympus BX61 fluorescence light microscope (Olympus, Tokyo, Japan). The antibodies used in this study are listed in Supplementary Table 1.

### Immunohistochemical studies of mouse tumour tissue

Frozen tissue sections (8 μm thick) of tumours were fixed with 4% paraformaldehyde solution in PBS. To detect endothelial cells, rat anti-CD31/PECAM-1 monoclonal antibody (1:100 dilution, MEC 13.3, BD Pharmingen, Franklin Lakes, NJ) was used. To determine the hypoxia of tumour tissue, rabbit anti-CA9 polyclonal antibody (1:1,000 dilution, Novus Biologicals, Littleton, CO) was used. Appropriate secondary antibodies conjugated with peroxidase (ready to use, Nichirei, Tokyo, Japan) and the 3,3′-diaminobenzidine tetrahydrochloride (DAB) Liquid System (DAKO, Carpinteria, CA) were used to detect the immunostaining. Nuclei were counterstained with hematoxylin (DAKO). MVD (the number of CD31^+^ structures per HPF) was evaluated at a × 200 magnification^15^,^53^,^66^. The CA9-positive areas were calculated using the Image J software program (National Institutes of Health, Bethesda, MD) at a × 200 magnification. Images were acquired by Keyence BZ-9000 microscopy (Keyence, Tokyo, Japan).

### Flow cytometry

The tumour tissues collected from Y-MESO-14 tumour-bearing SCID mice were suspended to provide single cells on day 28 after tumour cell-inoculation. To identify fibrocyte-like cells, PE-Cy7 conjugated antibodies to CD45 (1:50, 30-F11, eBioscience, San Diego, CA), Fluorescein conjugated anti-CXCR4 (1:10 dilution, 247506, R&D systems, Minneapolis, MN), and Biotin conjugated anti-COL1A1 (1:100 dilution, Rockland, Limerick, PA) were used. To detect Biotin conjugated antibody, PE-Streptavidin (1:1000 dilution, BD Pharmingen, Franklin Lakes, NJ) was used. FITC conjugated anti-CD45 (1:50 dilution, 30-F11, BD Pharmingen, Franklin Lakes, NJ) and PE conjugated antibodies to F4/80 (1:10 dilution, BM8, Biolegend, San Diego, CA) or Gr-1 (1:10 dilution, RB6–8C5, BD Pharmingen, Franklin Lakes, NJ) were used to detect macrophages or MDSCs, respectively. To determine the effect of AMD3100 on HSCs in bone marrow, the bone marrow cells were harvested from the tibias and femurs of tumour (Y-MESO-14)-bearing SCID mice on day 42 after the inoculation of tumour cells. The mice were treated once daily with AMD3100 (5 mg kg^−1^ per mouse) or vehicle control by subcutaneous injection from days 7–42, in addition to bevacizumab treatment. To identify HSCs, PE conjugated antibody to Sca-1 (1:10 dilution, D7, BD Pharmingen, Franklin Lakes, NJ) was used. The stained cells were analysed by flow cytometry using a BD LSRFortessa (BD Bioscience, San Diego, CA) for acquisition and FlowJo software (Treestar Inc, Ashland, OR) for analysis.

### Patient samples

Paraffin-embedded sections from 26 lung adenocarcinoma patients (surgery alone: *n*=11, chemotherapy without bevacizumab: *n*=5, chemotherapy with bevacizumab: *n*=10) were provided by The University of Tokushima (Tokushima, Japan), Fukui-ken Saiseikai Hospital (Fukui, Japan), Municipal Tsuruga Hospital (Fukui, Japan) and Kagawa University (Kagawa, Japan). Informed consent was obtained from all patients, and the protocol was approved by the Institutional Review Board (IRB) of Tokushima University Hospital (No. 1750) and then by the IRB of each of the participating institutions.

### Immunohistochemical studies of the patient samples

To detect fibrocyte-like cells in the clinical specimens, the paraffin-embedded tissues (4 μm thick) were stained with a rabbit anti-FSP-1 polyclonal antibody (1:100 dilution, Neomarkers/Lab Vision Corporation, Fremont, CA) and mouse anti-CD45 monoclonal antibody (1:50 dilution, 136-4B5, Cell Signaling Technology, Danvers, MA). To detect the FGF2-positive cells, a goat anti-FGF2 polyclonal antibody (1:50 dilution, Sigma, St Louis, MO) was used. We used a goat anti-IL-17 polyclonal antibody to detect Th17 cells (1:50 dilution, R&D systems, Minneapolis, MN). A mouse anti-CD68 monoclonal antibody (ready to use, PG-M1, Nichirei, Tokyo, Japan) was used to detect macrophages. A mouse anti-CD3 monoclonal antibody (1:50 dilution, F7.2.38, Dako, Carpinteria, CA) was used to detect T-cells. To detect the B cells, mouse anti-CD19 monoclonal antibody (1:50 dilution, LE-CD19, Dako, Carpinteria, CA) was used. These sections were re-incubated with appropriate secondary antibodies conjugated with peroxidase or alkaline phosphatase (ready to use, Nichirei, Tokyo, Japan). Immunoreactivity was detected by using the DAB Liquid System or new fuchsin (Nichirei, Tokyo, Japan), and samples were counterstained with hematoxylin. The number of positive cells was counted at a × 400 magnification. The expression levels of FGF2 in fibrocyte-like cells or macrophages were determined by performing double staining for FGF2 and FSP-1 or CD68, respectively, at a × 400 or × 800 magnification. Images were acquired by Keyence BZ-9000 microscopy (Keyence, Tokyo, Japan). To detect endothelial cells, CD31 immunohistochemistry was performed using Leica Bond-Max (Leica, Bannockburn, IL) and Bond Polymer Refine Detection (Leica, Bannockburn, IL) according to the manufacturer's protocols. A mouse anti-human CD31 monoclonal antibody (1:50 dilution, JC70A, Dako, Carpinteria, CA) was used as the primary antibody. To determine the microvessel length in tumour tissue, the major axis of vessels was measured by using the BZ-9000 analytical software program (Keyence, Tokyo, Japan).

### qRT-PCR

Total RNA was extracted from the tumours using an RNeasy Mini Kit (Qiagen, Valencia, CA), and was reverse-transcribed to cDNA using a High Capacity cDNA Reverse Transcription Kit (Applied Biosystems, Carlsbad, CA) according to the manufacturer's instructions. RT-PCR was performed using the CFX96 real-time PCR system (Bio-Rad, Hercules, CA) or the SYBR Premix Ex Taq (TAKARA, Kyoto, Japan). Human *RPL27* and mouse *RPS29* mRNA were used as housekeeping genes, and quantification was performed using the ΔΔ Ct method. The specific PCR primer pairs used for each studied gene are shown in Supplementary Table 2.

### Detection of protein expression in mouse tumour tissue

Y-MESO-14 tumour tissues were homogenized in T-PER (Pierce, Rockford, IL) containing phosphatase and protease inhibitor cocktails (Roche, Basel, Switzerland). The concentrations of proteins were determined using a Bio-Rad Protein Assay Kit (Bio-Rad, Richmond, CA). Samples of 500 μg ml^−1^ of total proteins and pleural effusion from tumour-bearing mice were used to detect the concentrations of human VEGF, mouse VEGF, FGF1 and FGF2 by using ELISA kits (R&D Systems, Minneapolis, MN) according to the manufacturer's instructions.

### Induction of chemical hypoxia *in vitro*

Y-MESO-14 tumour cells were plated in 24-well plates with 10% FBS containing DMEM and were incubated for 24 h. After the incubation, 100 μM of a hypoxia-mimetic, DFX mesylate (DFX, Sigma, St Louis, MO), was added to each well, and the cells were incubated for another 24 h to mimic the hypoxic condition. Total RNA was extracted from the hypoxic tumour cells and the gene expression levels were compared with those in tumour cells incubated under normoxic conditions.

### Isolation of human fibrocyte-like cells

Human fibrocyte-like cells were isolated according to previously published methods^67^. Mononuclear cells were isolated from the peripheral blood of healthy volunteers using Ficoll density centrifugation^68^. The isolated cells were cultured in DMEM supplemented with 20% FBS, penicillin and streptomycin on bovine fibronectin (R&D systems, Minneapolis, MN)-coated 150 mm cell culture dishes (BD Pharmingen, Franklin Lakes, NJ). The medium was changed twice a week. After 7–10 days, the media was aspirated and washed with sterile PBS three times. The adherent cells were determined to be circulating fibrocyte-like cells by a flow cytometric analysis and immunostaining^67^. Informed consent was obtained from all volunteers, and the protocol was approved by the IRB of Tokushima University Hospital (No. 1586).

### Purification of fibrocyte-like cells from mouse lung

Murine fibrocyte-like cells were isolated according to previously published methods^69^. Murine lungs removed from male SCID mice were minced with scissors and incubated with DMEM including 1 mg ml^−1^ BSA (Sigma, St Louis, MO), 1 mg ml^−1^ collagenase IV (Roche, Basel, Switzerland) and 100 μg ml^−1^ DNase 1 (Sigma, St Louis, MO) for 1 h at 37 °C. The single cell suspensions from whole lungs were incubated in DMEM supplemented with 20% FBS in 100 mm fibronectin-coated dishes. When cells reached 70% confluence, they were passaged using trypsin digestion. After 7 days, the trypsinized cells were incubated with anti-CD45 Abs coupled to magnetic beads (Miltenyi Biotech, Auburn, CA) for 15 min on ice. The labelled cells were separated into CD45^+^ (fibrocyte-like cells) and CD45^−^ (fibroblasts) populations using an Auto MACS instrument (Miltenyi Biotech, Auburn, CA) according to the manufacturer's instructions.

### Purification of tumour-infiltrated fibrocyte-like cells

The tumour tissues collected from Y-MESO-14 orthotopic tumour-bearing SCID mice were minced and suspended to provide single cells on day 28 after inoculation in the same way described above. The number of whole cells was counted using a hemocytometer, and 3 × 10^6^ suspended cells were labelled with anti-mouse CD45 magnetic beads, and the CD45^+^ cells were isolated using an Auto MACS instrument. Mouse CD45^+^ cells were plated in fibronectin-coated 100 mm-diameter dishes and incubated for seven days. The adherent cells were considered to be tumour-associated fibrocyte-like cells. The number of the cells was counted using a hemocytometer, and the total RNA was extracted.

### Cell migration assay for fibrocyte-like cells

Y-MESO-14 tumour cells were plated in 24-well plates (5 × 10^4^ cells per 500 μl per well) with 10% FBS containing DMEM and were incubated for 24 h. After the incubation, the medium was changed to 0.1% FBS containing DMEM with or without 100 μM DFX, and cells were incubated for another 24 h. The migration assay was performed using 8 μm pore size cell culture inserts (BD Biosciences, San Jose, CA). Purified human fibrocyte-like cells were plated in fibronectin-coated 100 mm-diameter dishes, and were incubated with DMEM supplemented with 10% FBS for 24 h. Then, the medium was changed to 0.1% FBS containing DMEM in the presence or absence of 5 μg ml^−1^ of the CXCR4 antagonist, AMD3100, and the cells were incubated for 1 h. After this incubation, the fibrocyte-like cells (3 × 10^5^ cells per 100 μl per well) were added to the inside of the cell culture inserts, and the inserts were put on the Y-MESO-14 tumour cells cultured under the conditions described above. After a 3 h incubation, the cells that had migrated to the bottom surface of the filter were stained using Diff-Quik reagents I and II (Baxter, Morton Grove, IL) and were counted in six randomly selected fields on each filter under a microscope at a × 200 magnification.

### Cell proliferation assay of HUVECs

Secondary cultures of human fibrocyte-like cells were plated into 24-well fibronectin-coated plates (1 × 10^5^ cells per well) with the culture medium described above. After 24 h, the medium was changed to 400 μl of 0.1% FBS containing DMEM, and the cells were incubated for an additional 48 h. The conditioned medium was collected and stored at −80 °C for further experiments. The proliferation of HUVECs was measured using the MTT (3-[4, 5-dimethylthiazol-2-yl]-2, 5-diphenyl tetrazolium) dye reduction method. HUVECs (2 × 10^3^ cells per 100 μl) were plated into each well of 96-well type 1 collagen coated-plates (Iwaki, Tokyo, Japan) in 0.1% FBS containing DMEM and were incubated for 24 h, then 100 μl of the culture supernatant from fibrocyte-like cells was added. Recombinant human VEGF protein (10 ng ml^−1^, R&D systems, Minneapolis, MN) or FGF protein (10 ng ml^−1^, PeproTech, Rocky Hill, NJ) -containing medium was added as a positive control. Then, bevacizumab (1 or 0.1 μg ml^−1^) or BGJ-398 (100 or 10 nM) was added, and the cells were incubated for an additional 72 h. After the incubation, 50 μl of stock MTT solution (2 mg ml^−1^, Sigma, St Louis, MO) was added to all of the wells, and the cells were incubated for 2 h at 37 °C. The media containing the MTT solution was removed, and the dark blue crystals were dissolved by adding 100 μl of DMSO. The absorbance was measured with a SUNRISE Remote R microplate reader (Tecan, Mannedorf, Switzerland) at test and reference wavelengths of 550 and 630 nm, respectively.

### Microarray analysis

Total RNA was extracted from snap-frozen Y-MESO-14 tumour tissues implanted in the SCID mice with or without bevacizumab treatment by using a RNeasy Mini Kit (Qiagen, Valencia, CA). The quality of the RNA samples were confirmed using an Agilent 2100 Bioanalyzer (G2938C, Agilent Technologies, Palo Alto, CA) employing an Agilent RNA 6000 Nano LabChip Kit (5067-1511, Agilent Technologies, Palo Alto, CA). A total of 200 ng of total RNA was amplified and labelled using the Agilent Low Input Quick Amp Labelling Kit, One-Color kit (5190-2305, Agilent Technologies, Palo Alto, CA), and labelled RNA was hybridized to Agilent Whole Human Genome Gene Expression 4 × 44 K ver. 2.0 Micro Arrays (Agilent Technologies, Palo Alto, CA) for 17 h at 65 °C. The Agilent Feature Extraction Image Analysis Software program (Version 10.7.3, Agilent Technologies, Palo Alto, CA) was used to extract data from the raw microarray image files. The data visualization and analysis were performed using the Gene Spring GX (Version 12.5, Agilent Technologies, Palo Alto, CA) software program. Genes with an altered expression with more than 2-fold were identified on each sample. The microarray data were deposited in the NCBI Gene Expression Omnibus (GEO) under accession code GSE59476.

### Statistical analyses

The data are presented as the means±s.e.m. The statistical analyses were performed using Student's *t*-test for unpaired samples, the Mann–Whitney-*U*-test or a one-way ANOVA, followed by Tukey's multiple-comparison *post-hoc* test, were also used as appropriate. Values of *P*<0.05 were considered to be statistically significant.

## Additional information

**Accession codes:** The microarray data were deposited in the NCBI Gene Expression Omnibus (GEO) under accession code GSE59476.

**How to cite this article:** Mitsuhashi, A. *et al*. Fibrocyte-like cells mediate acquired resistance to anti-angiogenic therapy with bevacizumab. *Nat. Commun.* 6:8792 doi: 10.1038/ncomms9792 (2015).

## Supplementary Material

Supplementary Information Supplementary Figures 1-12 and Supplementary Tables 1-2

## Figures and Tables

**Table 1:** Effects of bevacizumab on thoracic tumours in mice.

**Y-MESO-14**	**Thoracic tumour**		**Pleural effusion**	
	**Incidence**	**Weight (mg)**	**Incidence**	**Volume (μl)**
Control (*n*=4)	4/4	260 (210–330)	4/4	290 (200–700)
Bev 10 μg (*n*=5)	5/5	170 (100–250)*	4/5	50 (0–250)*

**PC14PE6**	**Lung**			**Pleural effusion**	
	**Weight (mg)**	**Metastasis**		**Incidence**	**Volume (μl)**
		**Incidence**	**Number**		
Control (*n*=7)	480 (320–880)	7/7	62 (25–87)	6/7	520 (0–750)
Bev 10 μg (*n*=7)	180 (170–200)†	6/7	17 (0–42)†	0/7	0
Bev 30 μg (*n*=7)	180 (150–250)†	7/7	29 (4–101)*	0/7	0

MPM, malignant pleural mesothelioma; SCID, severe combined immunodeficient

Y-MESO-14 (human MPM) cells were orthotopically injected to SCID mice, and PC14PE6 (human lung adenocarcinoma) cells were intravenously injected to nude mice. These mice were treated with bevacizumab (Bev) from day 7 after the tumour cell injection, and thoracic tumour, lung metastasis and pleural effusion were evaluated on day 28 (Y-MESO-14) or on day 35 (PC14PE6) after tumour cell injection. Values are the median (minimum–maximum).

^*^Statistically significant difference compared with control group (*P*<0.05) by the Mann–Whitney-*U*-test.

^†^Statistically significant difference compared with control group (*P*<0.01) by the Mann–Whitney-*U*-test.
