# Supplementary material for: Fibrocyte-like cells mediate acquired resistance to anti-angiogenic therapy with bevacizumab
Source: Nat Commun. 2015 Dec 4;6:8792. doi: 10.1038/ncomms9792 (PMC4686833; doi:10.1038/ncomms9792)
Supplement: Supplementary Information — Supplementary Figures 1-12 and Supplementary Tables 1-2 [file ncomms9792-s1.pdf]

Supplementary Figure 1

Bevacizumab resistance in microvessel density of EHMES-10 and PC14PE6 tumours.

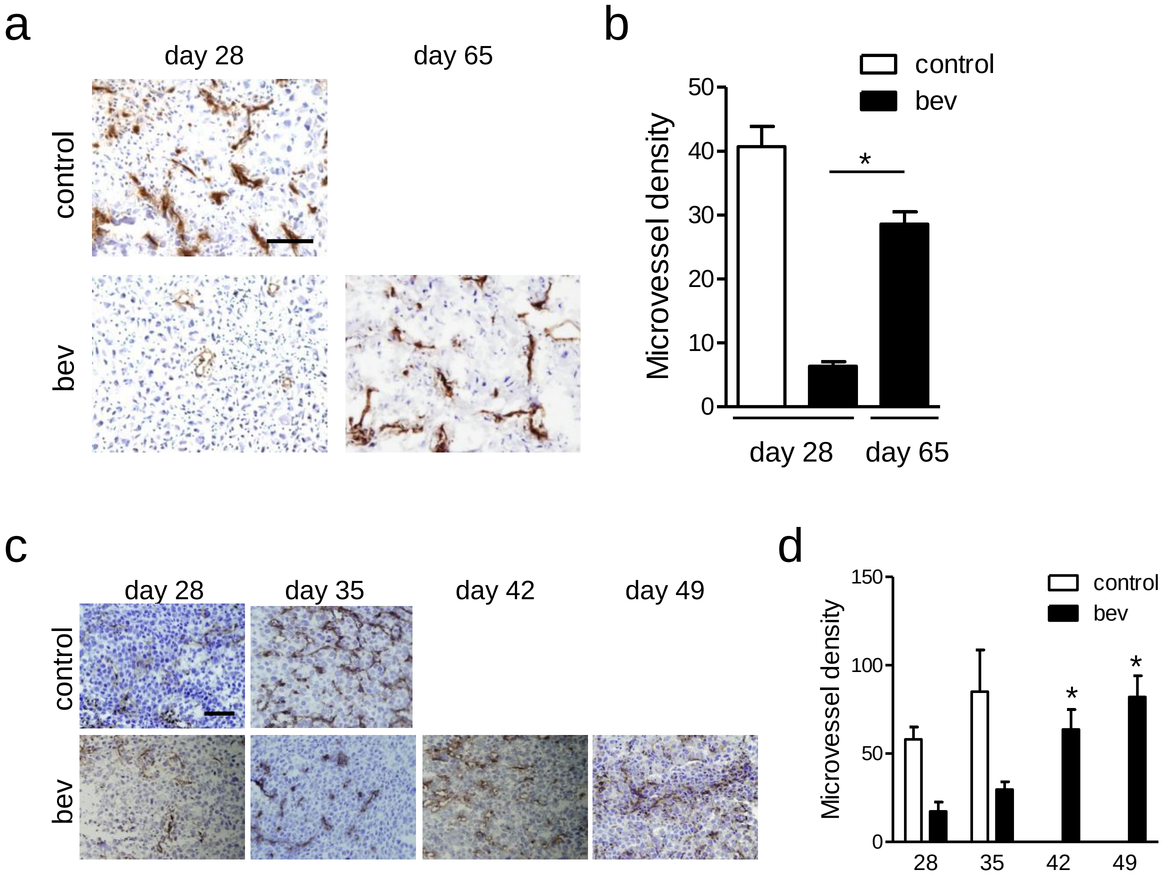

Supplementary Figure 1.

CD31 staining and the evaluation of microvessel density of the tumour produced by (a, b) EHMES-10 and (c, d) PC14PE6 cells. Mice were continuously treated with bevacizumab (bev) beginning seven days after tumour cell injection. (a, c) Representative images of sections from (a) EHMES-10 intrathoracic tumours and (c) PC14PE6 lung metastatic tumours stained for CD31. Tumours were harvested at different time points from both the control and bevacizumab-treated groups. Scale bar, 200 μm. (b, d) Microvessel density in each tumour (n=5 per group). The data are shown as the means ± s.e.m. *P<0.05 by a one-way ANOVA.

Supplementary Figure 2

Evaluation of tumour cell-derived pro-angiogenic factors in Y-MESO-14 tumours.

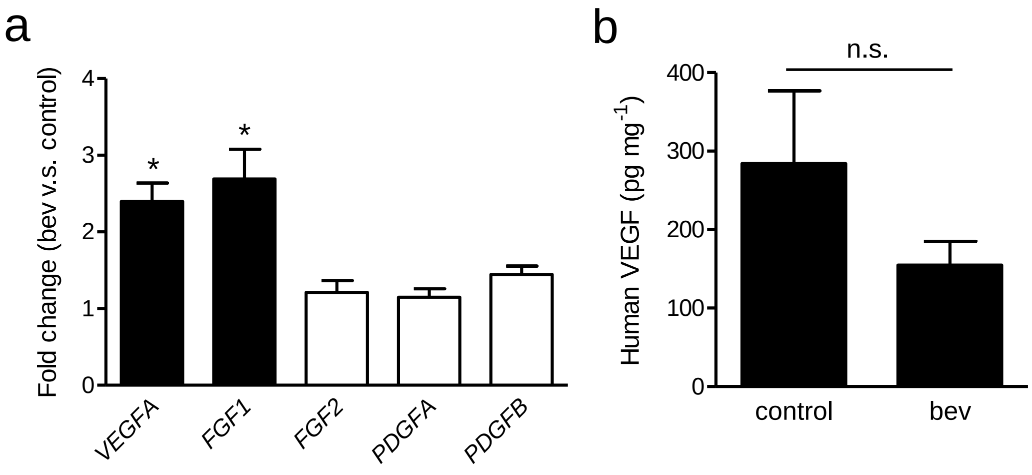

Supplementary Figure 2.

(a) A comparison of the tumour (human) mRNA expression of pro-angiogenic factors in the intrathoracic Y-MESO-14 tumours treated with or without bevacizumab (bev). The fold-changes in mRNA expression in the bevacizumab-treated tumours compared to control tumours determined by qRT-PCR are shown. **P*<0.05 by the Mann-Whitney-*U* test. (b) The amount of human VEGF measured by ELISA in Y-MESO-14 tumours treated with or without bevacizumab (n=4 per group). The data are shown as the means ± s.e.m. n.s.; not significant.

Supplementary Figure 3

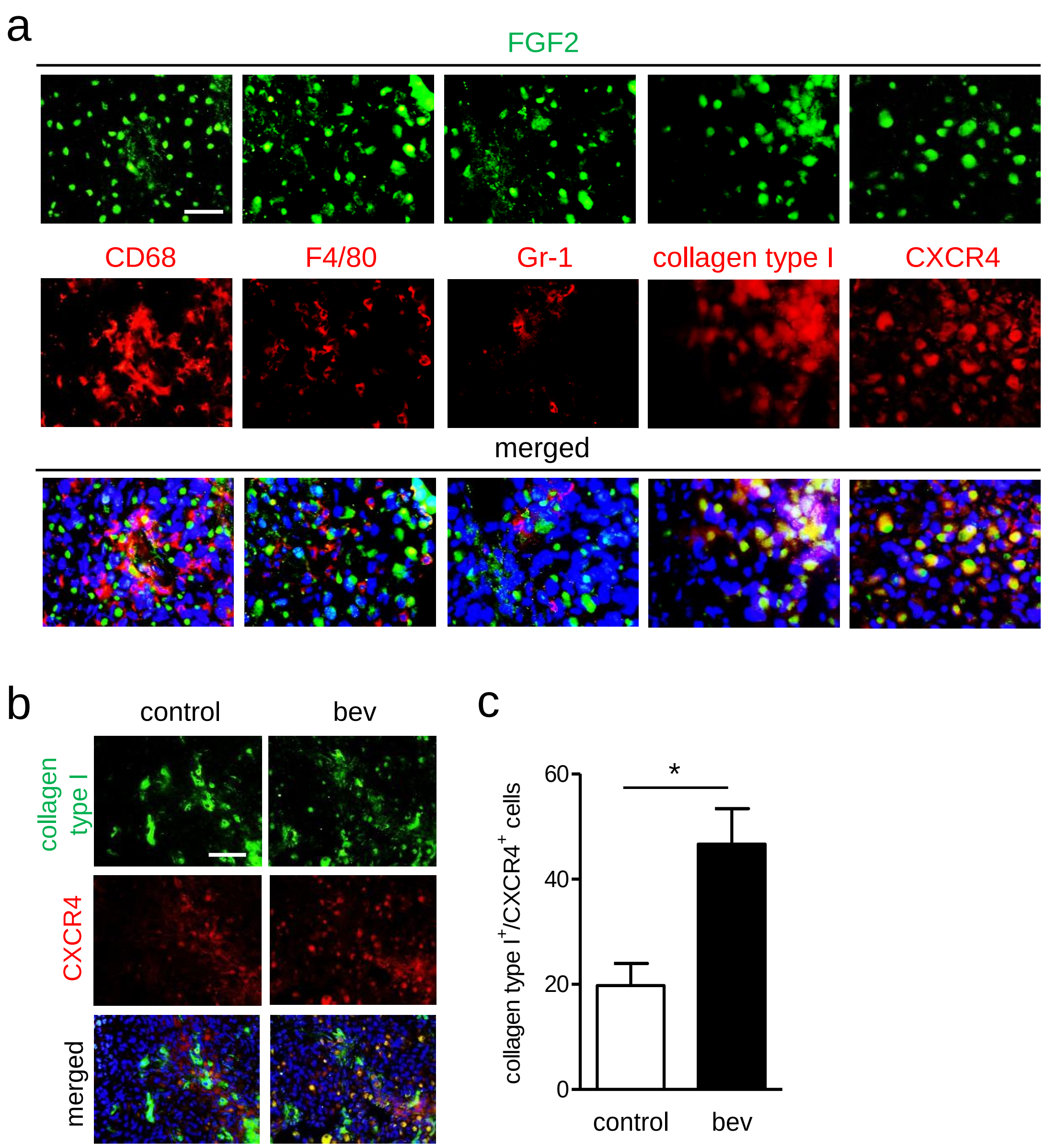

Supplementary Figure 3.

Identification of collagen type I$^+$/CXCR4$^+$ cells as FGF2-producing cells in the intrathoracic tumours produced by EHMES-10 cells. (a) Representative images of tumour sections from mice treated with bevacizumab. FGF2 (green) was co-stained with CD68, F4/80, Gr-1, collagen type I or CXCR4 (red). Scale bar, 100 μm. (b) Double staining for collagen type I and CXCR4 in the tumours (EHMES-10) from mice treated with or without bevacizumab (bev). Scale bar, 200 μm. (c) The number of double-positive cells in the tumours was compared between the control group and the bevacizumab-treated group (n=13 per group). *P<0.01 by the Mann-Whitney-*U* test. The data are shown as the means ± s.e.m.

Supplementary Figure 4

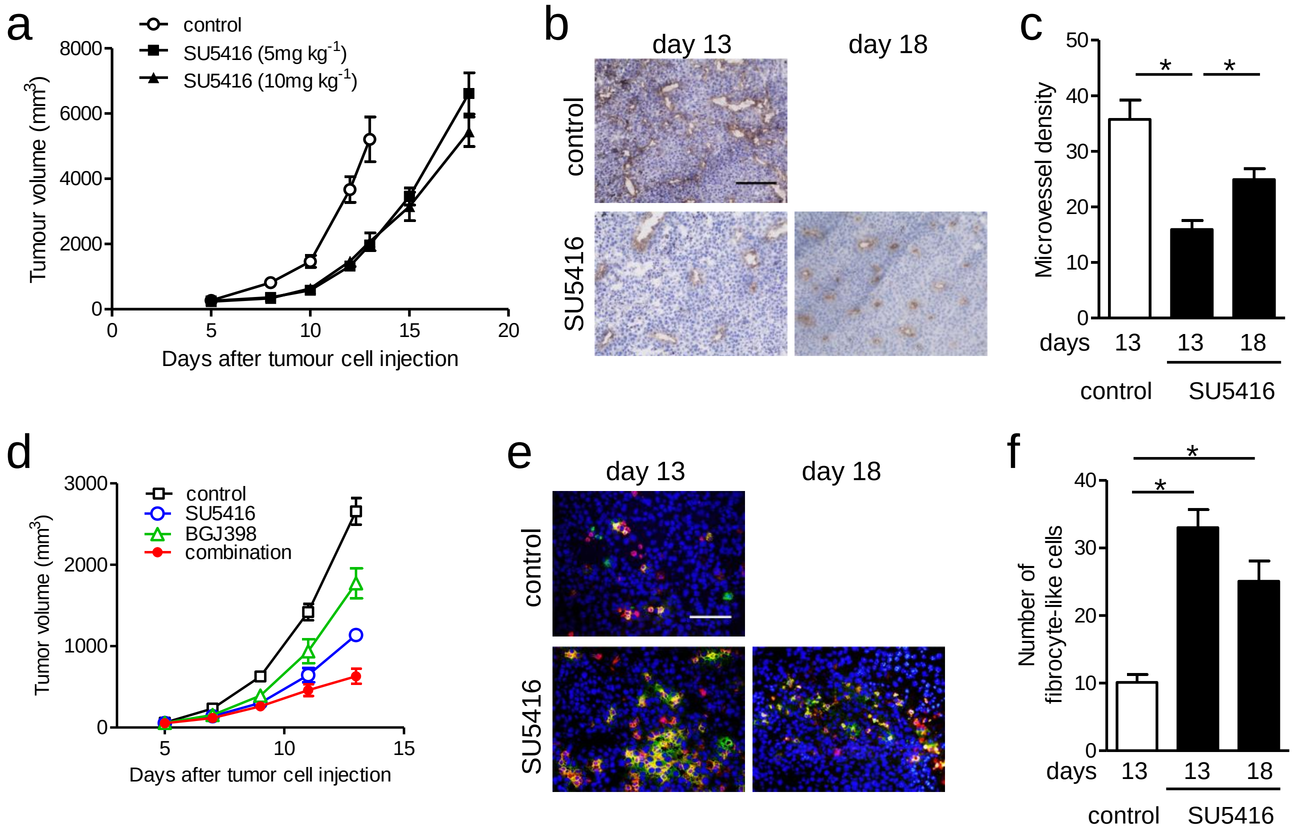

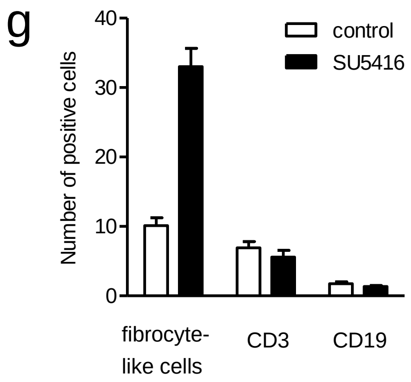

Supplementary Figure 4.

Assessment of the resistance against anti-VEGF therapy in syngeneic mouse model. B16 melanoma cells were subcutaneously injected to C57BL/6 mice, and the mice were continuously treated with daily SU5416, a VEGFR inhibitor, from day 5 after the tumour-cell injection. (a) Evaluation of the tumour volume. (b) Representative images of CD31 staining of the tumour harvested at different time points from the control- or SU5416-treated mice. Scale bar, 200 μm. (c) Microvessel density in each tumour (n=5-7 per group). The data are shown as the means ± s.e.m. *P<0.05 by a one-way ANOVA. (d) The tumour volume of the B16-bearing mice treated with SU5416, BGJ-398 (an FGFR inhibitor), or both. SU5416 and BGJ398 were continuously given from 5 days after tumour cell injection. (e) Representative images of double staining for FSP-1 (green) and CD45 (red) in the tumours from mice treated with or without SU5416. Scale bar, 200 μm. (f) The number of double-positive cells in the tumours was compared between the control group and the SU5416-treated group (n=19 per group). The data are shown as the means ± s.e.m. *P<0.05 by a one-way ANOVA. (g) Evaluation of the number of fibrocyte-like cells, $CD3^+$ or $CD19^+$ lymphocytes in the tumour of each treatment group harvested on day 13.

Supplementary Figure 5

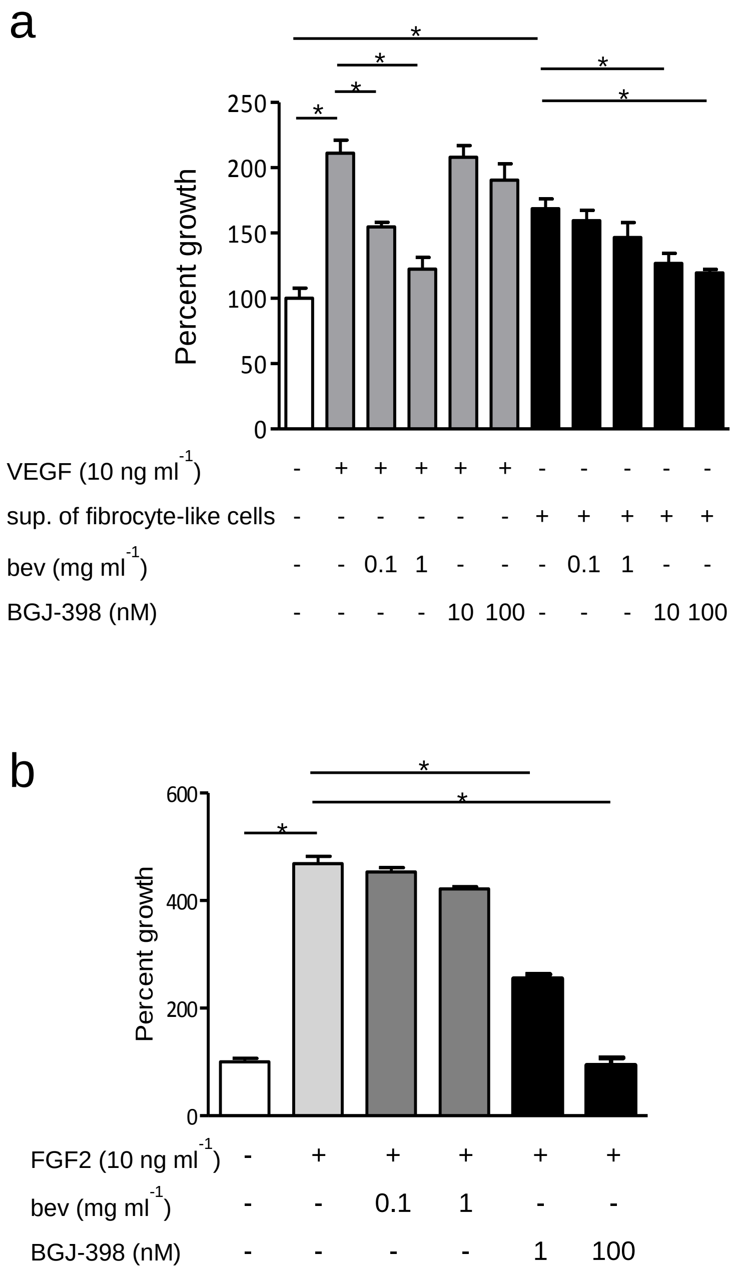

Supplementary Figure 5.

Effect of the culture supernatant of fibrocyte-like cells on the proliferation of HUVECs. The proliferation of HUVECs was measured by the MTT assay. The value obtained in the control group was set at 100%. (a) The culture supernatant of fibrocyte-like cells was collected after a 48-h incubation with 0.1% FBS. (b) The effect of recombinant FGF2 on the proliferation of HUVECs. The data are shown as the means ± s.e.m. *P<0.05 by a one-way ANOVA.

Supplementary Figure 6

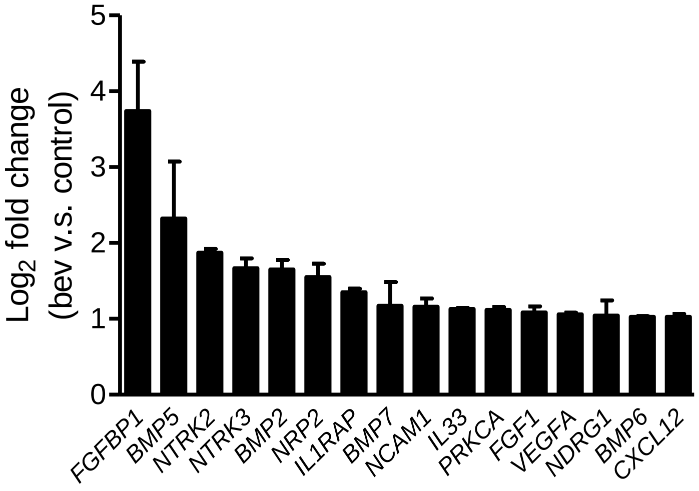

Supplementary Figure 6.

A microarray analysis was performed to compare the human (tumour cell) gene expression in the intrathoracic Y-MESO-14 tumours treated with or without bevacizumab (bev). The fold-changes in gene expression in the bevacizumab-treated tumours compared to the control tumours are shown. The data are shown as the means ± s.e.m.

Supplementary Figure 7

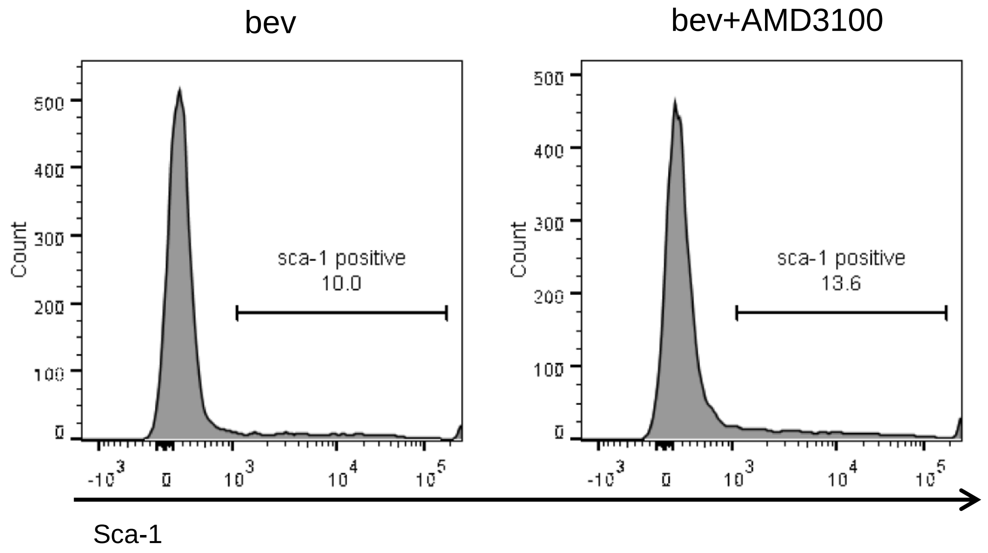

Supplementary Figure 7.

Flow cytometric analysis of Sca-1-positive HSCs in the bone marrow of tumour (Y-MESO-14)-bearing mice treated with bevacizumab (bev) or bevacizumab plus AMD3100. The bone marrow was harvested 42 days after tumour-cell inoculation. Data are the representative of two experiments with similar results.

Supplementary Figure 8

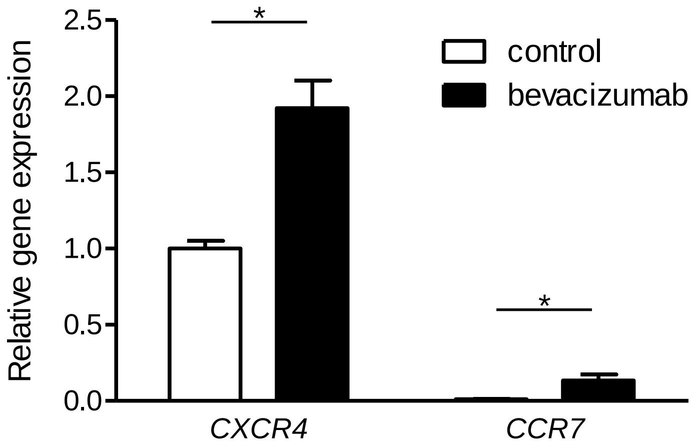

Supplementary Figure 8.

A comparison of the gene expression of stromal *CXCR4* and *CCR7* in the tumour produced by Y-MESO-14 cells. Tumours were harvested 28 days after the tumour cell-inoculation, and qRT-PCR analysis was performed to compare stromal *CXCR4* and *CCR7* expression. (n=5 per group). The data are shown as the means ± s.e.m. *P<0.01 by the Mann-Whitney-*U* test.

Supplementary Figure 9

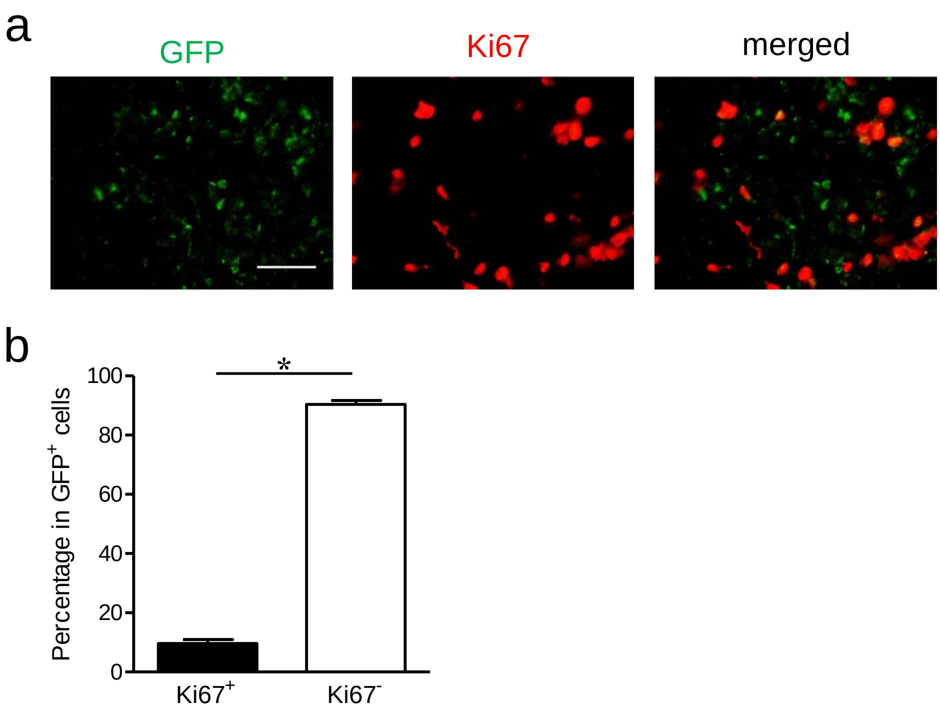

Supplementary Figure 9.

Assessment of the proliferating host-derived cells in bevacizumab-treated tumour in GFP bone marrow chimeric mouse model. (a) Representative images of tumour sections from GFP chimeric mice treated with bevacizumab. GFP (green) was co-stained with mouse-specific Ki67. Scale bar, 100 μm. (b) Among the GFP^{+} cells, the percentage of $Ki67^{+}$ cells were compared with the percentage of $GFP^{+}/Ki67^{-}$ cells (n=7 per group). The data are shown as the means ± s.e.m. *P<0.01 by the Mann-Whitney-*U* test.

Supplementary Figure 10

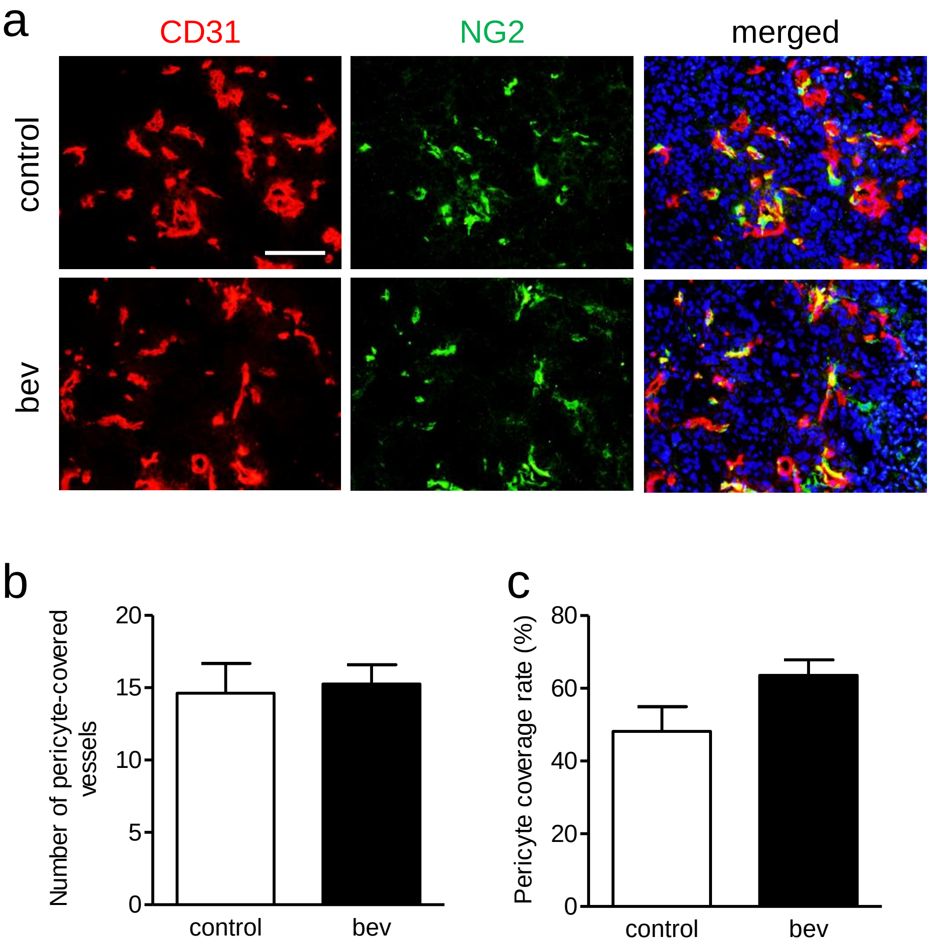

Supplementary Figure 10.

The pericyte coverage of microvessels in the intrathoracic tumours derived from Y-MESO-14 cells. (a) Representative images of double staining for CD31 (microvessels) and NG2 (pericytes) in Y-MESO-14 tumours treated with or without bevacizumab (bev). Tumours were harvested on day 35 after tumour cell injection (when tumours exhibited resistance to bevacizumab). Scale bar, 100 μm. (b) The number of pericyte-covered vessels (double positive for CD31 and NG2) and (c) the percentage of the coverage (number of pericytes covering vessels divided by the total microvessels) (n=13 per group). The data are shown as the means ± s.e.m.

Supplementary Figure 11

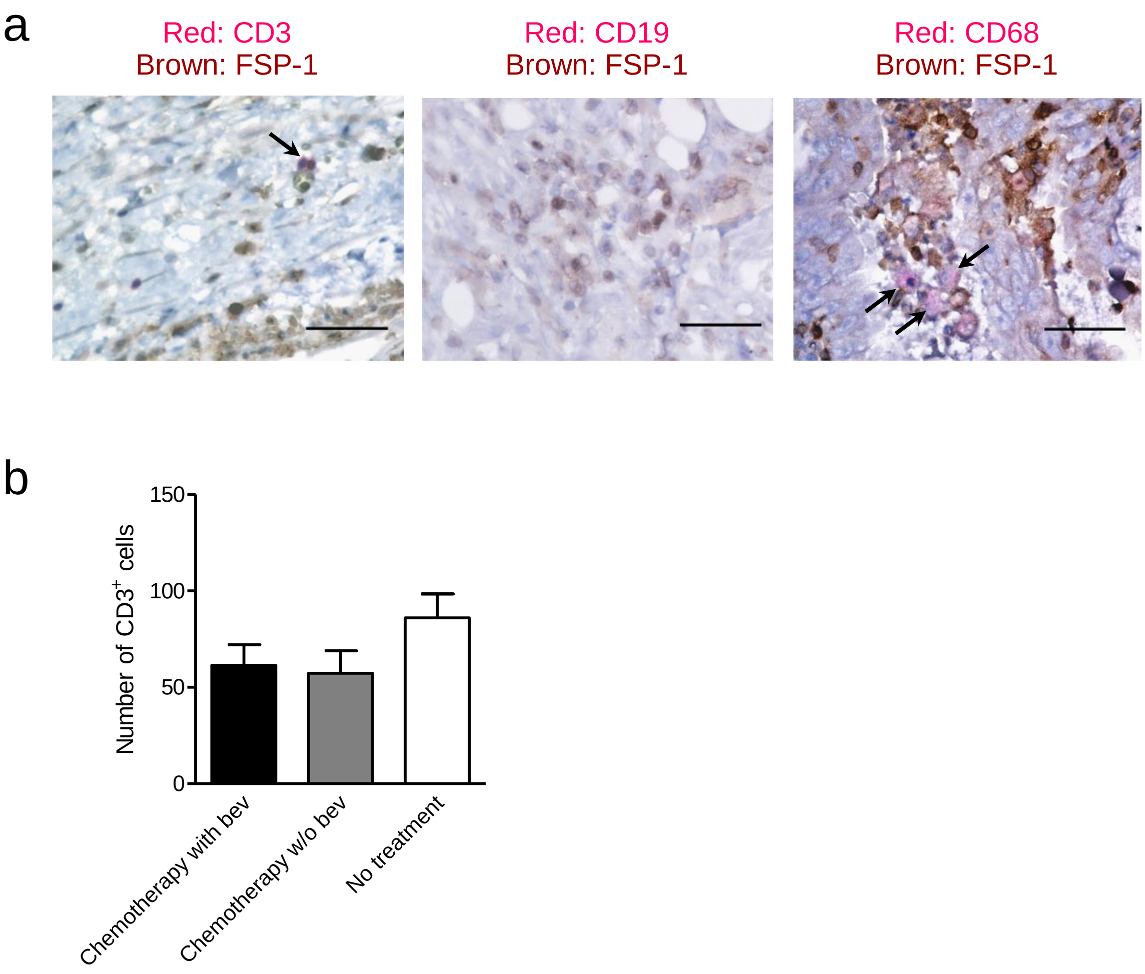

Supplementary Figure 11.

Immunohistochemistry of CD3, CD19, or CD68 co-stained with FSP-1 in the human lung cancer specimens. (a) Representative images of double staining of FSP-1 and CD3, CD19, or CD68 in the tumour tissues of patients treated with neoadjuvant chemotherapy containing bevacizumab. CD3, CD19, or CD68 was stained in red (indicated by the arrows), and FSP-1 was stained in brown. Scale bar, 100 μm. (b) The number of CD3-positive cells in tumour tissues did not change among the group of patients who received chemotherapy with bevacizumab, chemotherapy without bevacizumab, and no treatment (surgery alone). The data are shown as the means ± s.e.m.

Supplementary Figure 12

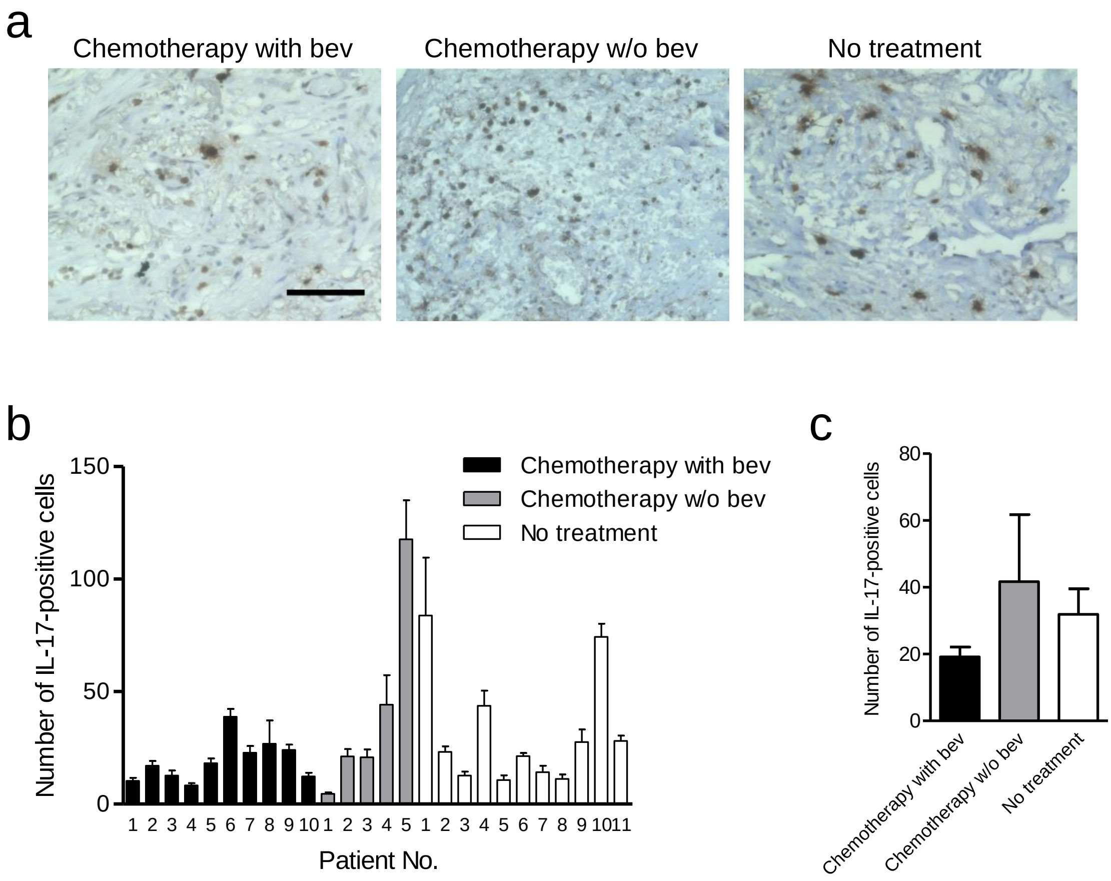

Supplementary Figure 12.

Assessment of the IL-17-expressing cells in surgically resected human lung cancer specimens. (a) Representative images of IL-17-positive cells (brown) in patients treated with neoadjuvant chemotherapy containing bevacizumab, chemotherapy alone (without bevacizumab) or no prior therapy. Scale bar, 100 µm. (b) The number of IL-17-positive cells in the individual tumour samples. (c) The average number of IL-17-positive cells in each group. The data are shown as the means ± s.e.m. There were no significant differences in the numbers of IL-17-positive cells among the groups.

Supplementary Table 1. The list of antibodies used.

Product	Sourse	Clone number	Dilution
**anti-mouse**			
goat anti-COL1A1 polyclonal antibody	Santa Cruz	-	1/100
Biotinylated rabbit anti-COL1A1 polyclonal antibody	Rockland	-	1/50
rabbit anti-FSP-1 polyclonal antibody	Neomarkers/Lab Vision Corporation	-	1/100
rat anti-CXCR4 monoclonal antibody	R&D systems	247506	1/100
FITC rat anti-CXCR4 monoclonal antibody	BD Pharmingen	2B11	1/25
rat anti-CD45 monoclonal antibody	BD Pharmingen	30-F11	1/50
FITC rat anti-CD45 monoclonal antibody	BD Pharmingen	30-F11	1/50
PE-Cy7 rat anti-CD45 monoclonal antibody	eBioscience	30-F11	1/50
rat anti-CD68 monoclonal antibody	Serotec	ED1	1/100
rat anti-F4/80 monoclonal antibody	Serotec	Cl:A3-1	1/100
PE rat anti-F4/80 monoclonal antibody	Biolegend	BM8	1/10
rat anti-Gr-1 monoclonal antibody	BD Pharmingen	RB6-8C5	1/50
PE rat anti-Gr-1 monoclonal antibody	BD Pharmingen	RB6-8C5	1/10
rat anti-CD3 monoclonal antibody	BD Pharmingen	17A2	1/150
PE rat anti-CD3 monoclonal antibody	BD Pharmingen	17A2	1/10
rat anti-CD19 monoclonal antibody	BD Pharmingen	1D3	1/150
PE rat anti-CD19 monoclonal antibody	BD Pharmingen	1D3	1/10
goat anti-αSMA polyclonal antibody	Abcam	-	1/150
rabbit anti-FGF2 polyclonal antibody	Santa Cruz	-	1/100
goat anti-FGF2 polyclonal antibody	Sigma	-	1/50
rat anti-CD31/PECAM-1 monoclonal antibody	BD Pharmingen	MEC 13.3	1/150
rabbit anti-NG2 polyclonal antibody	Millipore	-	1/150
rabbit anti-GFP monoclonal antibody	Cell Signaling Technology	D5.1	1/75
goat anti-GFP polyclonal antibody	Santa Cruz	-	1/50
rabbit anti-mouse Ki67 antibody	Cell Signaling Technology	D3B5	1/150
rabbit anti-CA9 polyclonal antibody	Novus Biologicals	-	1/1000
**anti-human**			
mouse anti-CD45 monoclonal antibody	Cell Signaling Technology	136-4B5	1/50
goat anti-IL-17 polyclonal antibody	R&D systems	-	1/50
mouse anti-CD68 monoclonal antibody	Nichirei	PG-M1	ready to use
mouse anti-CD3 monoclonal antibody	Dako	F7.2.38	1/50
mouse anti-CD19 monoclonal antibody	Dako	LE-CD19	1/50
mouse anti-CD31 monoclonal antibody	Dako	JC70A	1/50

Supplementary Table 2. The primer sequences used for qPCR

Genes (mouse)	Forward	Reverse	Product size (bp)
VEGFA	5´-GGAGATCCTTCGAGGAGCACTT-3´	5´-GGCGATTTAGCAGCAGATATAAGAA-3´	130
FGF1	5´-GACTTCATTCCCGTCTTGTG-3´	5´-TAGTTTCCTAGAGGCAGGTT-3´	166
FGF2	5´-CACGTCAAACTACAACTCCA-3´	5´-CGTCCATCTTCCTTCATAGC-3´	95
PDGFA	5´-GTGAGGTTAGAGGAACACCT-3´	5´-ATGTTCAGGAATGTCACACG-3´	148
PDGFB	5´-CAAGTGTGAGACAGTAGTGA-3´	5´-CATGGGTGTGCTTAAACTTTC-3´	158
VEGFR1	5´-CGTCACAGTCACCCTAAAAA-3´	5´-CTCTCCTACTGTCCCATGTT-3´	80
VEGFR2	5´-GTGGCGAAGATGTTTTTGAG-3´	5´-ACCGAAAGCAATAAAAGGCT-3´	150
VEGFR3	5´-CTACAACCTACAAAGCCTCC-3´	5´-TGCTTTCTAGCTCCTCAAAC-3´	84
FGFR1	5´-CCTACTTCTCCGTCAATGTC-3´	5´-GGTGTTGTCCGTCTCTTTCT-3´	98
FGFR2	5´-ATACGCCCACTCACTTTGCT-3´	5´-CTTTCTCCAATTCCAAAACACA-3´	80
PDGFRA	5´-AGGACTTGGGTGATGTGGAG-3´	5´-TGTGGCCAGTCACTCTCTTG-3´	176
PDGFRB	5´-TATACTATGCGAGCCTTCCA-3´	5´-AGGTAGACCAGGTGACATTT-3´	172
CXCR4	5´-TGGAACCGATCAGTGTGAGT-3´	5´-CATAGTCTCCAGAACCCACTT-3´	69
COL1A1	5´-CGCAAAGAGTCTACATGTCT-3´	5´-TTAGGCCATTGTGTATGCAG-3´	140
CD45	5´-AGTGCAAAGGAGACCCTATT-3´	5´-ATCACTGGGTGTAGGTGTTT-3´	143
RPS29	5´-TCTGAAGGCAAGATGGGTCA-3´	5´-CATTCAAGGTCGCTTAGTCC-3´	196
CCR7	5 ´ -GGCTAGCTGGAGAGAGACAAG -3´	5 ´ -AAATCACACAGGAAGGCTGT-3 ´	51
Genes (human)	Forward	Reverse	Product size (bp)
VEGFA	5´-TCTGCGCAGAGCACTTTG-3´	5´-CTCTAATCTTCCGGGCTCG-3´	142
FGF1	5´-CGTGGGGGAGGTGTATATAA-3´	5´-GGCCAACAAACCAATTCTTC-3´	182
FGF2	5´-CACCTATAATTGGTCAAAGTGG-3´	5´-CAGAAATTCAGTAGATGTTTCCC-3´	228
PDGFA	5´-AAGTCCAGGTGAGGTTAGAG-3´	5´-TGTCCTCTTCCCGATAATCC-3´	81
PDGFB	5´-AGCCTGTGGCTTGGAGTG-3´	5´-ACCCACCTAGAAGGGCAGTT-3´	125
CXCL12	5´-ACCGCGCTCTGCCTCA-3´	5´-CATGGCTTTCGAAGAATCGG-3´	76
RPL27	5´-ATCGCCAAGAGATCAAAGATAA-3´	5´-TCTGAAGACATCCTTATTGACG-3´	123
